# Long-term care insurance and multidimensional poverty of middle-aged and elderly: Evidence from China

**DOI:** 10.3389/fpubh.2023.1100146

**Published:** 2023-02-10

**Authors:** Wenxiu Li, Jin Ke, Fei Sun

**Affiliations:** ^1^School of Economics and Trade, Guangdong University of Finance, Guangzhou, China; ^2^School of Economics and Management, South China Normal University, Guangzhou, China; ^3^School of Social Work, Michigan State University, East Lansing, MI, United States

**Keywords:** long-term care insurance, multidimensional poverty, middle-aged and elderly, difference-in-differences model, China

## Abstract

**Introduction:**

This paper examined the impact of public long-term care insurance (LTCI) pilots in China on the multidimensional poverty status of middle-aged and older adults.

**Methods:**

Using panel data from the China Health and Retirement Longitudinal Survey, we utilized LTCI pilots conducted in different cities from 2012 to 2018 and assessed the impact of LTCI using a difference-in-differences strategy.

**Results:**

We found that the implementation of LTCI reduces the multidimensional poverty of middle-aged and older adults and their likelihood of future multidimensional poverty. LTCI coverage was also associated with a reduction in the likelihood that middle-aged and older adults in need of care fall into income poverty, living consumption poverty, health poverty, and social participation poverty.

**Discussion:**

From a policy perspective, the findings of this paper suggest that the establishment of an LTCI system can improve the poverty of middle-aged and older adults in several ways, which has important implications for the development of LTCI systems in China and other developing countries.

## 1. Introduction

The causes of poverty are complex and multifaceted, with one of the most overlooked being the need for care due to physical disability and handicap (1). According to research conducted in developing countries, people with disabilities and their families are poorer and more likely to fall into poverty than families without a disabled person (2–4). People with disabilities and their families, on average, face additional direct costs (e.g., medical expenses), indirect costs (e.g., informal care), and opportunity costs (e.g., labor supply) than families without a disabled person, making them more vulnerable to poverty or chronic poverty (5). The vast majority of care services are provided and paid for by families and individuals (6). Informal caregiving within the family can be extremely costly in terms of both time and money, and affect unpaid caregivers' physical and mental health (7). Primary caregivers' employment status and work hours may suffer as a result of caring for their parents (8). However, only 6% of the world's population has access to government-sponsored long-term care assistance (6). Skilled formal care from professional caregivers is expensive in both developed and developing countries. When the price of care is solely determined by the market, individuals in need of care can quickly fall into a poverty trap due to costly care. In 1995, Germany pioneered the world's first long-term care social insurance system based on the care assistance system, providing specialized long-term care insurance (LTCI), which has greatly alleviated the poverty of the disabled. In China, the first public LTCI program pilots began in 2016. The purpose of this study is to examine the poverty reduction effects of a public LTCI program recently piloted in China.

For China, the number of disabled older adults (including those with mild, moderate, and severe disabilities based on ADL scores) expected to rise from 43.75 million in 2020 to 91.4 million in 2050, with growth rates of 108, 104, and 120% for older adults with mild, moderate, and severe disabilities, respectively, in 2050 compared to 2020, and total care costs are expected to rise from ¥538 billion in 2020 to ¥8,530.8 billion in 2050 (9). The traditional model of informal care provided by families in China is unsustainable as the proportion of nuclear families rises. Family members are tethered to provide care for relatives with disabilities, making it difficult for them to find work and miss out on development opportunities, leaving families in financial distress. The most common type of poverty for people with disabilities and their families is “poverty due to disability.”

However, a single monetary indicator does not fully reflect the poverty status of the individual. Poverty does not only mean low income, but also a lack of individual capability. Sen (10) defines a person's capability as the combination of possible functioning that a person is capable of achieving, and functioning reflects a wide range of things or states that a person considers worth doing or achieving, including both the basic functions such as maintaining health, obtaining adequate nutrition, and avoiding disease, as well as complex functions such as happiness, self-esteem, and being able to participate in social activities. The loss of these functions is a manifestation of poverty and is closely related to progress in achieving social justice (11). Therefore, a multidimensional perspective is needed to investigate the capability deprivation of people with disabilities, and a multidimensional poverty theory based on the capability study approach provides this perspective. This theory contributes to the investigation of inequalities in the deprivation of capability among people with disabilities, as well as providing a comprehensive framework for investigating the negative effects suffered by people with disabilities (12). According to multidimensional poverty theory, income deprivation cannot be used as a consensual indicator variable to adequately capture the degree of individual deprivation in real-world contexts where markets are imperfect or non-existent. Individual deprivation must be considered in terms of multiple functional dimensions to properly measure individual poverty.

Previous studies have broadly selected dimensions from the following to measure the multidimensional poverty level of people with disabilities: Income, consumption, material wellbeing, social participation, health, psychological wellbeing, education, and employment are all factors to consider (4, 5, 12, 13). The additional direct, indirect, and opportunity costs that people with disabilities and their families must bear have a negative impact on their income and consumption (14). Difficulty in accessing basic life materials, such as sanitation and clean water, is also an important dimension of the capability deprivation of the poor. The physical mobility constraint of people with disabilities also leads to partial and complete loss of their social interaction and social mobilization, which often means weakened and discrete social networks, and the mobility constraint is an important cause of people's poverty (15). Furthermore, people with disabilities frequently face social exclusion in areas such as health care, education, employment, and social participation (16). People with disabilities tend to have poorer health and are more likely to be depressed compared to people without disabilities (17, 18).

The poverty alleviation system of the Chinese government has been based on the multidimensional poverty theory, which focuses on five aspects of poverty alleviation: food, clothing, education, medical care, and housing. The “poverty alleviation by improving health care” policy is designed to protect the people in poverty the right to health, preventing poverty from being caused by illness, and preventing poverty from returning due to illness. In the context of this policy, to alleviate the “poverty due to disability” problem, the number of LTCI pilot cities in China increased from Qingdao to 49 cities from 2012 to 2021, with over 140 million participants. At the moment, individual contributions, financial subsidies, and the transfer of existing public health insurance fund balances are the primary sources of funding for the LTCI fund. For people with varying degrees of disability, the LTCI program offers a variety of care service plans and medical care coverage. The LTCI program offers three types of services: home care, institutional care, and hospital care, with home care offering two types of services: basic life care and usual clinical care.

For those who already receive LTCI services, LTCI may contribute to poverty reduction in multiple dimensions. For the household income dimension, LTCI has an impact on household income through two main impact paths. The first pathway is that LTCI can reduce care and medical expenditures. Lu et al. (19) find that LTCI led to significant reductions in individual hospital care and medical expenditures in Qingdao. Feng et al. (20) show that LTCI led to significant reductions in individual hospital care and medical expenditures in Shanghai. Kim and Lim (21) suggest that subsidies for formal care can reduce expenditures for the least able elderly in Korea. The reduction in care and medical expenses has increased household income. The second pathway is that LTCI can reduce the care burden and increase the labor supply of family caregivers. In Japan, LTCI reduces the burden on family caregivers and has a significant positive spillover effect on the labor supply to family caregivers (22, 23). Similarly, Shinya and Nakamura (24) find that LTCI can increase the female labor supply by reducing the burden of informal caregiving. The reduced burden of family caregiving and the increased labor supply of informal caregivers brought about an increase in family income. Rudiger and Seiler (25) find that LTCI causes a change in the income of some people who needed long-term care, causing them to no longer require social assistance. For the living consumption dimension, LTCI has a positive effect on living consumption (i.e., living expenses including clothing, food, housing, transportation, entertainment, communication, etc., excluding medical and educational expenses). In the same way that other public health insurance has a consumption-boosting effect (26), LTCI can promote living consumption by reducing care and medical expenditures and increasing household income. Similarly, on the material wellbeing dimension, LTCI can improve material wellbeing (e.g., sanitation, clean water) by lowering care and medical expenditures and increasing household income. For the health, psychological wellbeing, and social participation dimensions, previous studies on LTCI in developed countries found that it is associated with improved health status and lower mortality among those who are covered (27, 28). The diverse service formats of Chinese public LTCI programs may also help improve the health status and psychological wellbeing of the population covered by LTCI. Lei et al. (29) find that LTCI coverage improved the health status of the covered population in China and reduced mortality, as well as improved depression. Improvements in health status and psychological wellbeing may also increase the frequency of social activities (30). Care services provided by LTCI may also help increase the frequency of social activities (31). For those who have not yet received LTCI services but are enrolled in LTCI, LTCI may promote living consumption and improve material wellbeing by reducing the anticipated financial burden from expected long-term care needs (32). Furthermore, LTCI may improve their health outcomes by alleviating the psychological burden associated with anticipated long-term care needs or the caregiving burden on the current care recipient's family caregiver (29).

The potential poverty-reducing efforts of Chinese public LTCI programs remain unknown. In this study, we examine the poverty reduction efforts of LTCI on middle-aged and older adults covered by the program, which are of particular interest to policy makers. Many developing countries that have plans to implement LTCI can also benefit from the experience of China's LTCI pilot.

Using panel data from the China Health and Retirement Longitudinal Survey (CHARLS), this paper contributes to the literature in the following ways. First, few studies have linked LTCI to poverty and focused on the impact of LTCI on poverty. With the perspective of multidimensional poverty, we assess the multidimensional poverty status of middle-aged and older adults across six dimensions: income, living consumption, material wellbeing, health, psychological wellbeing, and social participation, and evaluate the impact of LTCI on the multidimensional poverty status of middle-aged and older adults using a difference-in-differences (DID) strategy based on LTCI pilots launched in different cities in China from 2012 to 2018. We also analyze the heterogeneity of the impact of LTCI on multidimensional poverty based on the differences in LTCI systems in each pilot city to provide useful references for optimizing the LTCI system in China in more detail. Second, in order to more comprehensively assess the poverty reduction effect of LTCI, we construct multidimensional poverty vulnerability (MPV), a forward-looking indicator of poverty, and evaluate the impact of LTCI on it. Third, as one of the few studies evaluating the impact of the LTCI pilot, this paper provides evidence that the implementation of LTCI reduced multidimensional poverty and multidimensional poverty vulnerability among middle-aged and older adults. These findings are important for a complete cost-benefit analysis of LTCI and have policy relevance for other middle-income countries and developing countries.

## 2. Institutional background

**Figure 1:**
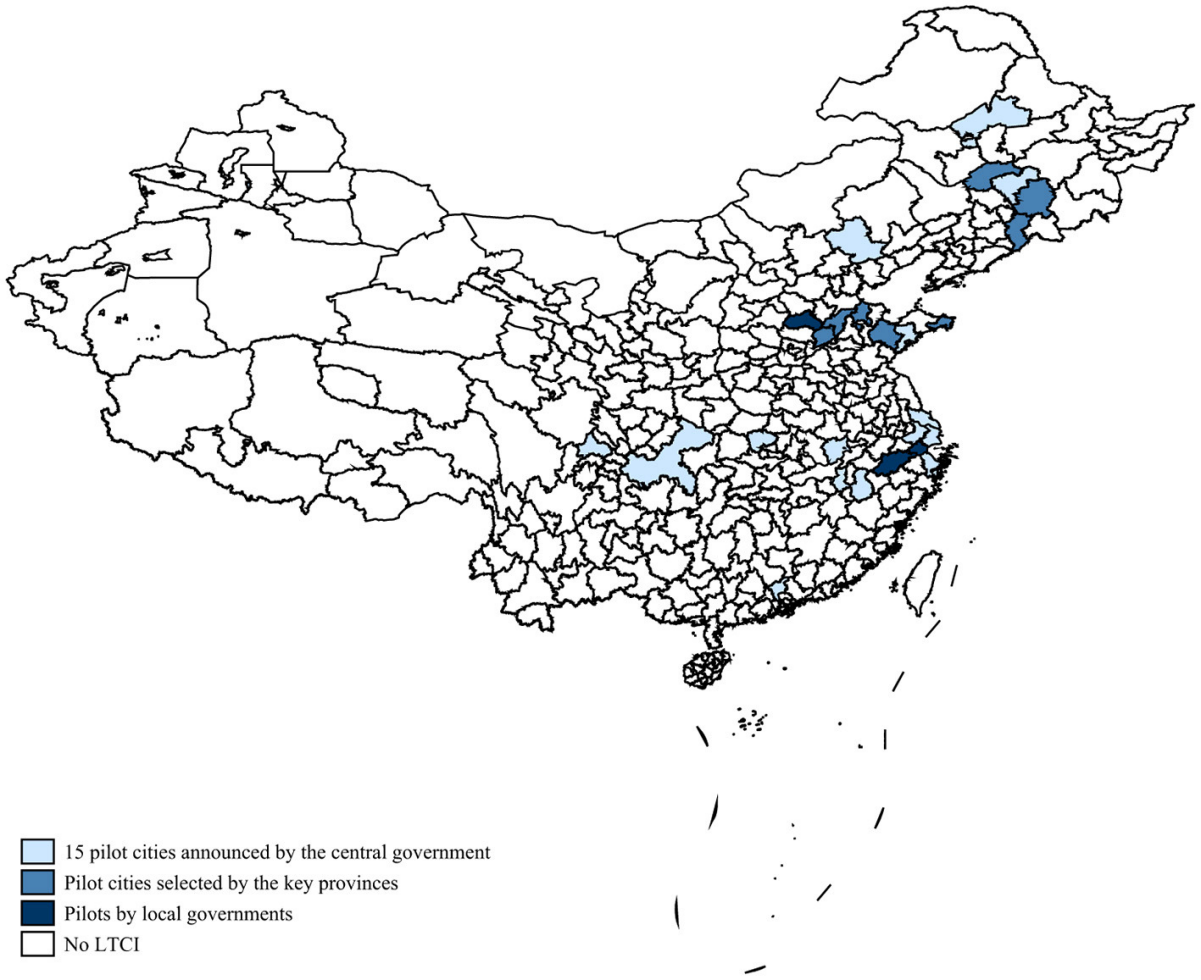
LTCI pilot cities (China, 2012-18). Self-collected by the author from publicly available policy documents at all levels of government. For details, see Appendix B.

To ensure affordable long-term care services for people in need, the Chinese central government issued guidelines in July 2016 to establish an LTCI system, conducted the first public LTCI pilots in 15 cities, and identified two provinces (i.e., Jilin and Shandong Provinces) as key provinces, which can choose cities within their provinces (other than the 15 cities mentioned above) as LTCI pilots.^1^ Individual cities among the 15 initial pilot cities (e.g., Qingdao and Changchun) had previously launched LTCI pilots, and Weifang in Shandong Province had also launched an LTCI pilot in January 2015. In addition to the 15 first LTCI pilot cities and the self-defined LTCI pilot cities of the two key provinces, individual cities (e.g., Jiaxing) conducted their LTCI pilots in 2016–2017 (see Figure 1).

The LTCI program relies on three basic medical insurance programs in China to expand its coverage: the Urban Employee Basic Medical Insurance (UEBMI), the Urban Resident Basic Medical Insurance (URBMI), and the Urban-Rural Resident Basic Medical Insurance (URRBMI). URBMI and New Rural Cooperative Medical Insurance (NRCMI) merged to form URRBMI, but URBMI remains in some cities. In 2020, UEBMI, URBMI, and URRBMI have covered more than 95% of the population. Residents who are already enrolled in basic health insurance will likely be automatically enrolled in LTCI, depending on the actual situation in the pilot cities.

The LTCI programs in most pilot cities were funded by basic medical insurance funds, and in some cities, the pilot programs were also supported by financial subsidies (e.g., Suzhou, Nantong) or welfare lottery funds (e.g., Shihezi). Individual cities may require individual contributions (e.g., Anqing requires an annual individual contribution of 20 RMB) or employer contributions (e.g., Shangrao requires an employing unit contribution of 5 RMB per employee per year) for funding.

Most cities require at least 6 months of long-term severe disability to receive benefits, but some cities (e.g., Nantong, Suzhou) allow people with moderate disabilities to receive LTCI benefits. The criteria for disability assessment are also not consistent. The majority of pilot cities assessed disability using the Barthel ADL index, while some pilot cities used localized assessment criteria (e.g., Suzhou, Chengdu).

Home care, full-time institutional care, and full-time hospital care are the three main types of services provided by the LTCI program. LTCI's expense reimbursement rates are all above 70%. In most cases, the actual treatment payment rate for people receiving LTCI benefits is usually determined by the service type. Home care benefits are covered at a higher rate than institutional or hospital care (e.g., LTCI pays 90% of the cost for those who choose home care in Guangzhou, with a payment ceiling of up to 115 RMB/person/day. However, LTCI pays 75% of the cost for those who choose institutional care, with a payment ceiling of up to 120 RMB/person/day.).

Although the LTCI program has only been in place for a short time, it is rapidly expanding in China. By 2020, there were 108,353,000 LTCI participants, among whom 835,000 people received benefits. There were 4,845 LTCI designated care service institutions, and 191,000 care service personnel. In 2020, LTCI fund income was 19.61 billion yuan (or $2.9 billion), fund expenditure was 13.14 billion yuan (or $1.93 billion), and per capita subsidies were 15,700 RMB (or $2,310). On top of the original LTCI pilot cities, the Chinese central government expanded the LTCI pilot cities in May 2020, adding 14 new cities as LTCI pilots, making every province in the country have at least one LTCI pilot city. In September of that year, the Chinese central government increased the number of LTCI pilot cities to 49 and announced a plan to create a policy framework for a unified LTCI system by 2025.

## 3. Data and variables

### 3.1. China Health and Retirement Longitudinal Survey

This study used secondary data from the China Health and Retirement Longitudinal Survey (CHARLS), a large interdisciplinary survey project hosted by Peking University's National Development Institute, implemented by Peking University's China Social Science Survey Center, and funded by the National Institute on Aging in the United States, the World Bank, and the National Natural Science Foundation of China (34). CHARLS aims to collect individual and household-level data on the Chinese population aged 45 and over, as well as their spouses. In 2011, CHARLS conducted a national baseline survey, and respondents were followed up every 2 years. CHARLS conducted survey interviews in 150 counties and 450 communities (or villages) in 28 provinces (autonomous regions and municipalities directly under the central government) across China in 2013, 2015, and 2018, with a total of 19,000 respondents in 12,400 households covered by 2018. Zhao et al. (34) provided more detailed information on the CHARLS sampling procedure, data quality, attrition, and response rates.

We primarily used CHARLS data from 2011, 2013, 2015, and 2018 (the latest available) for the following reasons. First, the main beneficiaries of LTCI are middle-aged and elderly people, and its poverty reduction effort is primarily reflected in the elderly group, and CHARLS is a survey data specifically for it over 45 years old and their spouses, which meets the research criteria of this paper. Second, this paper focuses on the poverty reduction effort of LTCI, and the CHARLS questionnaire contains typical questions that can represent each dimension of poverty, providing data support for this study. Third, in this study, Qingdao, the first city subject to the LTCI policy intervention, began the pilot in 2012, and subsequent cities began the pilot at various points in time. The four data waves allow the difference-in-differences (DID) approach to be used to study the differences before and after the policy pilot's implementation. The use of a household follow-up survey format for CHARLS also facilitates control for unobservable variables. Finally, our primary study sample is a four-period unbalanced panel of 36,439 surveyed middle-aged and older adults (11,162 individuals in 2011, 10,441 in 2013, 7,801 in 2015, and 7,035 in 2018) from 123 cities. The mean age of the sample was 61 years (SD = 9.226) and the median age was 60 years, which corresponds to the beneficiary population of LTCI.

### 3.2. Outcome variables

#### 3.2.1. Index of multiple deprivation

Poverty is a multidimensional concept. Measuring poverty from a multidimensional perspective is necessary not only to more accurately assess the breadth and depth of poverty, but also to provide better targeted and effective disaggregated relief measures for the poor (35).

The “dual cut-off” approach proposed by Alkire and Foster (36) is the current mainstream approach to measuring multidimensional poverty. The first cut-off determines whether the sample is deprived in each dimension, while the second cut-off determines whether the sample is in multidimensional poverty status based on the number of dimensions in which the sample is deprived. The method specifically measures multidimensional poverty through the following steps.

In the first step, let represent the *n*×*m* dimensional matrix and let the matrix element *y*∈*W*^*n, m*^ represent the values obtained by *n* individuals in *m* different dimensions. Any element *y*_*ij*_ in *y* represents the value taken by an individual *i* in dimension *j*, *i*∈(1, 2, …, *n*), *j*∈(1, 2, …, *m*). The row vector *y*_*i*_ = (*y*_*i*1_,*y*_*i*2_,…, *y*_*im*_) includes the values taken by individual *i* in all dimensions. Similarly, the column vector *y*_*j*_ = (*y*_1j_, *y*_2j_, …, *y*_*nj*_) represents the distribution of values taken by different individuals in dimension *j*.

The second step is to discern the poverty status of individuals on unidimensional and multidimensional dimensions. Let *Z* = (*Z*_1_, *Z*_2_, …, *Z*_*m*_) be the deprivation threshold matrix, and denote by *Z*_j_ (*Z*_j_>0) the threshold value at which an individual is deprived in the dimension *j*. If the welfare level of individual *i* in dimension *j* is less than the deprivation threshold *Z*_j_, the individual is judged to be poor under dimension *j*. The discriminative process is shown below:


(1) $$G_{\text{ij}}^{\partial }\{\begin{array}{c} {\left(\frac{z_{\text{j}}-y_{\text{ij}}}{z_{\text{j}}}\right)}^{\partial },y_{\text{ij}}<z_{\text{j}}\\ 0,y_{\text{ij}}\geq z_{\text{j}} \end{array}$$

When ∂ takes a value of 0, the matrix $G_{\text{ij}}^{\partial }$ discriminates whether individual *i* is in poverty in dimension *j* (i.e., when $G_{\text{ij}}^{\partial }=1$, it means that individual *i* is in poverty in dimension *j*, and the opposite when $G_{\text{ij}}^{\partial }=0$). When ∂ = 1 and *y*_*ij*_<*z*_*j*_, $G_{ij}^{\partial }$ can represent the proportional gap in deprivation suffered by individual *i* in dimension *j*.

Further introducing the determination of individual multidimensional poverty, each element of the matrix $G_{ij}^{\partial }$ represents the poverty status of individual *i* in a single dimension, defining *k* as the deprivation dimension threshold used to identify whether individual *i* is in multidimensional poverty, *C*_*i*_ as the multidimensional deprivation score of individual *i*,$C_{i}=\sum_{j=0}^{k}w_{j}G_{ij}^{0}$. *w*_*j*_ is the weight of dimension *j* in the multidimensional poverty measure, which indicates the relative importance of each dimension. In general, the “dual cut-off” method uses the dimensional equal weight method. When *C*_*i*_≥*k*, individual *i* is judged to be in multidimensional poverty, but not in it if the opposite is true.

The third step is to calculate the index of multiple deprivation (IMD). The index of multiple deprivation *IMD* and average deprivation share *A* are calculated as follows:


(2) $$\begin{array}{l} A=\sum_{i=1}^{n}C_{i}\left(k\right)/qm \end{array}$$

(3) $$\begin{array}{l} H=q/n \end{array}$$

(4) $$\begin{array}{l} IMD=\sum_{i=1}^{n}C_{i}\left(k\right)/nm \end{array}$$

*q* denotes the number of people in multidimensional poverty at the deprivation dimension threshold *k*. *C*_*i*_(*k*) denotes the value of *C*_*i*_ at the deprivation dimension threshold *k*. The incidence of poverty is *H*, *A* denotes the average deprivation share, and *IMD* is the index of multiple deprivation. Given the deprivation dimension threshold *k*, the index of multiple deprivation *IMD* is determined by both the incidence of poverty *H* and the average deprivation share *A* (i.e.,*IMD* = *H*×*A*).

#### 3.2.2. Construction dimensions of IMD

We identified 6 dimensions and 11 indicators to measure the index of multiple deprivation: income (one indicator), consumption (one indicator), material wellbeing (four indicator), social participation (one indicator), health (two indicator), psychological wellbeing (two indicators; see Table 1).

**Table 1:** Dimensions of poverty and indicators of deprivation.

**Dimension**	**Indicators**	**Deprivation cut-off**
Income	Annual household income per capita	Annual per capita income below the absolute poverty line set by the central government (based on the constant price of 2300 RMB in 2011) = 1; else = 0
Living consumption	Household per capita daily living expenses	Household per capita daily living expenses below World Bank daily living consumption Poverty Line = 1; else = 0
Material wellbeing	Cooking fuel	Use of straw, firewood or coal as the main domestic fuel = 1; else = 0
	Drinking water	Non-tap water or filtered water for drinking or cooking = 1; else = 0
	Flushing toilet	Toilet can't flush = 1; else = 0
	Household living area per capita	Household living area per capita is < 12 square meters = 1; else = 0
Social participation	Social activity	No social activity in the past month = 1; else = 0
Health	Self-reported health	Self-reported health is fair or bad = 1; else = 0
	Chronic disease	Have a chronic disease = 1; else=0
Psychological wellbeing	Life satisfaction	Life satisfaction is not very satisfied or not at all satisfied = 1; else = 0
	CES-D score	CES-D depression self-assessment score ≥ 20 = 1; else = 0

Source: Zhao et al. (33).

(1). The CHARLS questionnaire inquired about the number of people living in each household as well as the total annual household income. Total annual household income includes both wage and investment income from household members. Pensions, wages, and allowances make up annual wage income. After-tax income from industrial and commercial projects, rent, income from financial assets (e.g., funds, stocks, etc.), and income from agriculture comprise investment annual income. Divide the total annual household income by the number of household members to get the annual per capita household income. The threshold is determined by the central government's absolute poverty line (measured in constant 2011 prices). (2) The World Bank daily living consumption poverty line is $1.25 (2005 PPP) in 2011–2013 and $1.90 (2011 PPP) in 2015–2018. The World Bank daily living consumption poverty line is used as the deprivation cutoff for household per capita daily living expenses in this paper. Household living expenses are the sum of various household expenses such as clothing, food, housing, transportation, communication, and entertainment, but exclude medical and education expenses. (3) The Chinese government does not currently have a uniform standard for the minimum guaranteed housing area per capita, and the minimum guaranteed housing area per capita in each city varies between 10 and 15 square meters. We use 12 square meters as the deprivation cut-off for Household living area per capita. Household living area per capita is calculated by dividing the total household living area by the number of people living in the house. (4) The CHARLS questionnaire includes the Center for Epidemiological Survey, Depression Scale (CES-D). According to the scale, an individual is considered to have depressive symptoms if the CES-D score is ≥ 20. Therefore, we set the CES-D score ≥20 as the deprivation cut-off.

We chose indicators for measuring multidimensional poverty based on data availabilities, comparability, and representativeness. Comparability implies that dimension selection must be comparable across countries or internationally, and representativeness requires that indicator selection can represent the main characteristics of local poverty (37).

The first dimension selected is income. An assessment of the effectiveness of multidimensional poverty alleviation in China shows that long-term poverty alleviation targeting net income per capita contributes significantly to long-term multidimensional poverty alleviation among Chinese rural residents as a whole (38). This suggests that income continues to have a significant impact on poverty in rural China.

The second dimension is identified as living consumption. Living consumption takes on a different meaning compared to income in China, where the welfare coverage for the population is relatively limited compared to those in welfare states (e.g., universal health care) and where there is a tradition of high savings rates, as people often save on living expenses to cope with uncertainty. Moreover, living consumption is closely related to individual welfare, but monetary income does not guarantee a specific consumption basket due to imperfections in market functioning and consumer preference patterns (39). Therefore, in the Chinese context, it is necessary to include the living consumption dimension in a multidimensional poverty measurement system.

Four indicators from the material wellbeing dimension are included in our study. Cooking fuel, sanitation, and drinking water are all important issues for rural China's poverty, and these three indicators meet the official United Nations International Children's Emergency Fund (UNICEF) definition of an acceptable sanitation threshold. People who suffer from indoor air pollution as a result of cooking with wood, straw, or coal, do not have access to safe drinking water, and do not have access to flushable toilets are classified as poor. Our measure of material wellbeing also includes household living space per capita. One indicator of poverty in urban China is crowded living spaces.

The fourth dimension is health. To assess the health dimension, we used two indicators: self-rated health and the presence of chronic diseases. Health is a basic capability, and good health is linked to human development and social justice (40). In China, poverty caused by disease accounts for up to 40% of the poor population. Improving the health of the poor also helps them to escape from poverty through their work and reduces the likelihood of them falling back into poverty due to illness.

Finally, we included an indicator of social participation: whether they had engaged in social activities (e.g., socializing with friends, playing mahjong, chess, cards, or going to the community room) in the past month. People with disabilities who need care may face difficulties with social exclusion and participation in social activities (41, 42). Furthermore, social exclusion may have an impact on the mental health of people with disabilities (43). As a result, we included the psychological wellbeing dimension, which we measured using two indicators: life satisfaction and CES-D depression self-assessment scores.

We used the dimensional equal-weighting approach used in most studies, with the weights of each dimension set to be equal (i.e., the indicator weights under each dimension account for 1/6), and the weights of each indicator contained within the same dimension are also equal. Furthermore, there is no defined criterion for multidimensional poverty thresholds, so we set the multidimensional poverty threshold at 1/3 based on the experience of most studies (44, 45), which means we use *k* = 1/3 to calculate the IMD. Furthermore, if more than half of the indicators in each dimension of multidimensional poverty are poor, we consider the individual to be poor in that dimension and assign a value of 1, otherwise 0. The IMD structure constructed in this paper and the sensitivity analysis of the impact of different weighting structures on IMD are detailed in Appendix A.

**Figure 2:**
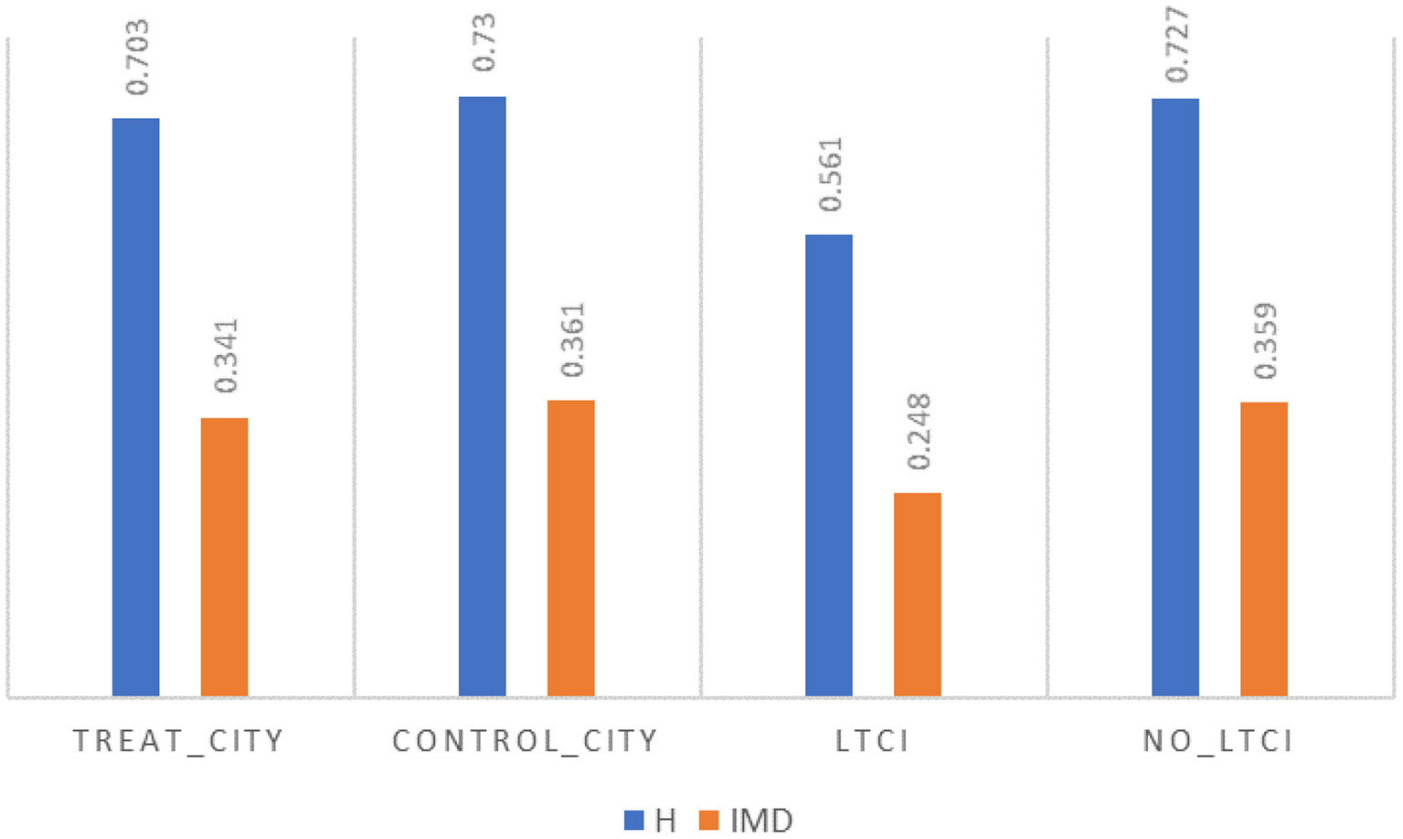
Decomposition of MPI by study design.

We decomposed the IMD according to the two classification criteria of treatment group cities, control group cities, and whether or not they are covered by LTCI within the treatment group cities according to the research design of this paper. Poverty incidence is ~3% lower in the treatment group cities than in the control group cities, as shown in Figure 2, but this may be due to the non-randomized and selective nature of the policy pilot, and thus this concern must be mitigated in subsequent robustness tests. The incidence of poverty (*H*) among those already covered by LTCI is much lower than among those not covered by LTCI, but the magnitude of the LTCI poverty reduction effect and whether the existence of this effect is robust and plausible still needs further empirical testing.

#### 3.2.3. Measurement of multidimensional poverty vulnerability

Poverty vulnerability, like poverty, should be a multidimensional concept and structure, and estimates of poverty vulnerability based only on the economic dimension are an important limitation to the scope of poverty (46). Azeem et al. (47) discovered that ex post economic poverty indicators are more prone to bias in identifying poor groups, and it is necessary to broaden the discussion beyond economic poverty to include multidimensional poverty and multidimensional poverty vulnerability. Multidimensional poverty vulnerability is a forward-looking indicator that depicts the dynamics of multidimensional poverty by measuring the likelihood of future multidimensional poverty among individuals. Feeny and McDonald (48) used a feasible generalized least squares (FGLS) method to estimate the MPV of Melanesian households and discovered that MPV is more prevalent than multidimensional poverty.

We use the feasible generalized least squares (FGLS) method to estimate MPV based on Feeny and McDonald (48). A high vulnerability to multidimensional poverty indicates that individuals are still highly likely to experience multidimensional poverty again in the future. We estimate the MPV using the following estimator:


(5) $$\begin{array}{l} {\overset{\wedge }{V}}_{MPI_{i}}=\mathrm{Pr}\left(MPI_{i}>k|X_{i}\right)=\Phi \left(\frac{X_{i}{\overset{\wedge }{\beta }}_{FGLS}-k}{\sqrt{X_{i}{\overset{\wedge }{\theta }}_{FGLS}}}\right) \end{array}$$

where ${\overset{\wedge }{V}}_{MPI_{i}}$ is the estimated probability value of the occurrence of multidimensional poverty for individual *i* in the future, IMD is the multidimensional deprivation score of individual *i*, Φ is the normal distribution cumulative density function, $X_{i}{\overset{\wedge }{\beta }}_{FGLS}$ and $X_{i}{\overset{\wedge }{\theta }}_{FGLS}$ are expected and fluctuating consistency estimates of multidimensional poverty for individuals in the future period, respectively.

### 3.3. Key independent variables

The first independent variable is LTCI coverage, which measures whether or not individuals are enrolled in LTCI. It was determined based on the policy document, which indicates when the pilot city started the LTCI pilot and who was covered in that city. Our dataset includes individuals surveyed in 15 LTCI pilot cities, 12 of which are the first batch of LTCI pilot cities identified by the central government. The LTCI programs in all 15 pilot cities cover groups that are already enrolled in UEBMI, with four covering URRBMI enrollees and one covering coverage for URBMI enrollees. LTCI coverage is an independent variable with intervention coded as 1 and not covered as 0. Appendix B contains specific information on when the LTCI pilot cities launched the program and which groups were served.

The duration of LTCI coverage (i.e., the difference between the survey interview date and the LTCI project start date in each pilot city) is the second independent variable, coded as 0 if the individual was not covered by LTCI.

### 3.4. Descriptive statistics

Table 2A presents descriptive statistics for the entire sample, treatment and control group. The *t*-test indicates the level of significance of the comparison between groups. The results of the simple DID estimation for each outcome variable are given in the last column. The simple DID analysis showed that individuals in the treatment group showed improvements in multidimensional poverty, income poverty, living consumption poverty, material wellbeing poverty, social participation poverty, health poverty, and psychological wellbeing poverty.

**Table 2A:** Summary statistics (2011–2018 panel).

	**Full**	**Treatment group**		**Control group**				***ΔΔ***
		**(LTCI pilot cities)**		**(non-LTCI pilot cities)**				
		**Pre-treat**	**Post-treat**	**2011**	**2013**	**2015**	**2018**	
Observations	36,439	4,034	107	11,162	10,441	7,801	7,035	
**Outcome variables**								
Index of multiple deprivation								
IMD	0.418 (0.167)	0.406 (0.166)	0.336 (0.151)^***^	0.384 (0.162)	0.423^***^ (0.169)	0.446^***^ (0.158)	0.432^***^ (0.172)	−0.067^***^ [0.024]
Single dimensional poverty (poverty = 1; otherwise = 0)								
Income poverty	0.521 (0.500)	0.481 (0.500)	0.112^***^ (0.317)	0.418 (0.493)	0.540^***^ (0.498)	0.720^***^ (0.449)	0.438^***^ (0.496)	−0.120^***^ [0.038]
Living consumption poverty	0.345 (0.475)	0.330 (0.470)	0.196^***^ (0.399)	0.281 (0.450)	0.381^***^ (0.486)	0.319^***^ (0.466)	0.422^***^ (0.494)	−0.161^*^ [0.089]
Material wellbeing poverty	0.717 (0.450)	0.755 (0.430)	0.897^***^ (0.305)	0.647 (0.478)	0.594^***^ (0.491)	0.644 (0.479)	0.680^***^ (0.466)	−0.074 [0.045]
Social participation poverty	0.638 (0.481)	0.614 (0.487)	0.607 (0.491)	0.651 (0.477)	0.745^***^ (0.436)	0.724^***^ (0.447)	0.772^***^ (0.419)	−0.052 [0.054]
Health poverty	0.726 (0.446)	0.729 (0.445)	0.710 (0.456)	0.733 (0.442)	0.717^***^ (0.450)	0.713^***^ (0.453)	0.742 (0.437)	−0.066 [0.052]
Psychological wellbeing poverty	0.154 (0.361)	0.139 (0.346)	0.131 (0.339)	0.175 (0.380)	0.147^***^ (0.354)	0.124^***^ (0.330)	0.166^*^ (0.372)	0.002 [0.038]
**Individual demographic covariates**								
Age	61.075 (9.226)	60.971 (8.967)	64.421^***^ (8.691)	59.090 (9.483)	60.871^***^ (9.303)	62.012^***^ (9.034)	63.408^***^ (8.154)	
Gender (male = 1; female = 0)	0.481 (0.500)	0.480 (0.500)	0.542 (0.501)	0.482 (0.500)	0.479 (0.500)	0.481 (0.500)	0.484 (0.500)	
Marital status (married = 1; unmarried, divorced and widowed = 0)	0.869 (0.338)	0.880 (0.325)	0.879 (0.328)	0.876 (0.330)	0.872 (0.334)	0.861^***^ (0.346)	0.862^***^ (0.345)	
Hukou status (urban residents = 1; rural residents = 0)	0.181 (0.385)	0.203 (0.402)	0.598^***^ (0.493)	0.227 (0.419)	0.202^***^ (0.401)	0.134^***^ (0.341)	0.129^***^ (0.336)	
Level of education								
No formal education (illiterate) = 1	0.255 (0.436)	0.229 (0.420)	0.112^***^ (0.317)	0.255 (0.436)	0.259 (0.438)	0.272^***^ (0.445)	0.230^***^ (0.421)	
Did not finish primary school but capable of reading or writing = 2	0.193 (0.395)	0.232 (0.422)	0.178 (0.384)	0.175 (0.380)	0.182 (0.386)	0.201^***^ (0.401)	0.229^***^ (0.420)	
Sishu/home school =3	0.004 (0.061)	0.003 (0.059)	—	0.005 (0.071)	0.004 (0.062)	0.004 (0.064)	0.001 (0.029)	
Graduate from elementary school = 4	0.227 (0.419)	0.239 (0.427)	0.252 (0.436)	0.221 (0.415)	0.223 (0.416)	0.234^**^ (0.423)	0.238^**^ (0.426)	
Graduate from middle school = 5	0.208 (0.406)	0.196 (0.397)	0.168 (0.376)	0.214 (0.410)	0.210 (0.407)	0.197^***^ (0.398)	0.211^***^ (0.408)	
Graduate from high school = 6	0.075 (0.263)	0.071 (0.257)	0.187^***^ (0.392)	0.080 (0.271)	0.078 (0.268)	0.066^***^ (0.247)	0.072^***^ (0.259)	
Graduate from vocational school =7	0.022 (0.146)	0.024 (0.153)	0.075^***^ (0.264)	0.027 (0.161)	0.025 (0.155)	0.018^***^ (0.133)	0.014^***^ (0.115)	
Graduate from two/three year college/associate degree = 8	0.012 (0.107)	0.005 (0.068)	0.028^***^ (0.166)	0.017 (0.130)	0.015 (0.121)	0.007^***^ (0.081)	0.004^***^ (0.062)	
Graduate from 4 year college/bachelor's degree = 9	0.005 (0.068)	0.001 (0.027)	—	0.008 (0.086)	0.005^**^ (0.072)	0.002^***^ (0.047)	0.002^***^ (0.040)	
Full-time employment (have full-time job = 1; no full-time job = 0)	0.668 (0.471)	0.667 (0.471)	0.383^***^ (0.488)	0.687 (0.464)	0.672^**^ (0.470)	0.694 (0.461)	0.603^***^ (0.489)	
Have basic old-age insurance	0.651 (0.477)	0.649 (0.477)	0.935^***^ (0.248)	0.286 (0.452)	0.812^***^ (0.391)	0.764^***^ (0.425)	0.863^***^ (0.344)	
Retirement support								
Relying on children for retirement = 1	0.655 (0.475)	0.579 (0.494)	0.140^***^ (0.349)	0.669 (0.471)	0.635^***^ (0.481)	0.673 (0.469)	0.643^***^ (0.479)	
Relying on savings for retirement = 2	0.039 (0.193)	0.038 (0.190)	0.009 (0.097)	0.041 (0.199)	0.042 (0.201)	0.042 (0.202)	0.027^***^ (0.161)	
Relying on pension or retirement salary for retirement = 3	0.255 (0.436)	0.325 (0.468)	0.822^***^ (0.384)	0.229 (0.420)	0.283^***^ (0.450)	0.243^**^ (0.429)	0.264^***^ (0.441)	
Relying on commercial pension insurance for retirement = 4	0.004 (0.060)	0.003 (0.057)	—	0.005 (0.069)	0.003^*^ (0.058)	0.004 (0.060)	0.002^***^ (0.048)	
Relying on other for retirement = 5	0.048 (0.215)	0.055 (0.228)	0.028 (0.166)	0.056 (0.230)	0.037^***^ (0.189)	0.039^***^ (0.193)	0.064^**^ (0.244)	
Have UEBMI	0.099 (0.298)	0.050 (0.217)	0.579^***^ (0.496)	0.117 (0.322)	0.117 (0.321)	0.072^***^ (0.258)	0.072^***^ (0.259)	
Have URBMI	0.043 (0.203)	0.066 (0.248)	0.159^***^ (0.367)	0.045 (0.208)	0.052^**^ (0.221)	0.042 (0.200)	0.028^***^ (0.165)	
Have URRBMI	0.037 (0.190)	0.093 (0.290)	0.262^***^ (0.442)	0.012 (0.111)	0.020^***^ (0.142)	0.019^***^ (0.138)	0.122^***^ (0.327)	
Hospitalization (hospitalized in the past year =1; else = 0)	0.129 (0.335)	0.126 (0.332)	0.168 (0.376)	0.091 (0.287)	0.129^***^ (0.335)	0.146^***^ (0.354)	0.171^***^ (0.376)	
Log (inpatient out-of-pocket expenses + 1)	0.293 (1.545)	0.238 (1.400)	0.331 (1.525)	0.151 (1.110)	0.268^***^ (1.478)	0.377^***^ (1.741)	0.462^***^ (1.934)	
**Household-level covariates**								
Log (household debt + 1)	1.421 (3.523)	1.031 (3.040)	1.197 (3.451)	0.811 (2.733)	1.914^***^ (3.962)	1.600^***^ (3.706)	1.457^***^ (3.594)	
Number of household members	3.502 (1.847)	3.345 (1.682)	2.262^***^ (0.757)	3.590 (1.818)	4.978^***^ (1.738)	2.517^***^ (1.165)	2.262^***^ (0.759)	
Number of household durables	4.603 (2.184)	4.685 (2.183)	5.140^**^ (2.230)	4.246 (2.161)	4.928^***^ (2.340)	4.679^***^ (2.097)	4.601^***^ (1.982)	
Household education andand training expenses	1.344 (2.985)	1.253 (2.888)	1.510 (3.335)	0.380 (1.560)	2.024^***^ (3.499)	1.543^***^ (3.174)	1.642^***^ (3.266)	

Standard deviations are in parentheses. The last column clusters standard errors at the city level, which are listed in square brackets. t-tests are used to compare pre-treatment and post-treatment within the treatment group cities, as well as a pairwise comparison of individuals in the control group cities in 2011 with 2013, 2015, and 2018.

* *p* < 0.10.

** *p* < 0.05.

*** *p* < 0.01.

To account for potential confounders, we included age, gender, marital status, hukou status (i.e., registered residence status), level of education, full-time employment, basic old-age insurance, retirement support, three indicators of health insurance, hospitalization status, and inpatient out-of-pocket expenses in the demographic and socioeconomic covariates. Furthermore, we controlled for several household-by-year variables, including household debt, number of household members, number of household durables, and household education & training expenses. As shown in Table 2A, the post-treatment population in treatment group cities was slightly older, more urban residents, had higher levels of education, higher levels of household capital, fewer people with full-time jobs, preferred to use pensions or retirement salaries for retirement, had smaller families, and tended to have basic pension insurance as well as the three types of basic medical insurance. Table 2B presents the characteristics of each variable studied.

**Table 2B:** Descriptive statistics (full sample).

**Variables**	***N***	**Mean**	**SD**	**Min**	**Med**	**Max**
**Outcome variables**						
Index of multiple deprivation						
IMD	36,439	0.418	0.167	0	0.417	1
Single dimensional poverty (poverty = 1; otherwise = 0)						
Income poverty	36,439	0.521	0.500	0	1	1
Living consumption poverty	36,439	0.345	0.475	0	0	1
Material wellbeing poverty	36,439	0.717	0.450	0	1	1
Social participation poverty	36,439	0.638	0.481	0	1	1
Health poverty	36,439	0.726	0.446	0	1	1
Psychological wellbeing poverty	36,439	0.154	0.361	0	0	1
**Individual demographic covariates**						
Age	36,439	61.08	9.226	45	60	108
Gender	36,439	0.481	0.500	0	0	1
Marital status	36,439	0.869	0.338	0	1	1
Hukou status	36,439	0.181	0.385	0	0	1
**Level of education**						
No formal education (illiterate)	36,439	0.255	0.436	0	0	1
Did not finish primary school but capable of reading or writing	36,439	0.193	0.395	0	0	1
Sishu/home school	36,439	0.00400	0.0610	0	0	1
Graduate from elementary school	36,439	0.227	0.419	0	0	1
Graduate from middle school	36,439	0.208	0.406	0	0	1
Graduate from high school	36,439	0.0750	0.263	0	0	1
Graduate from vocational school	36,439	0.0220	0.146	0	0	1
Graduate from 2/3 year college/associate degree	36,439	0.0120	0.107	0	0	1
Graduate from 4 year college/bachelor's degree	36,439	0.00500	0.0680	0	0	1
Full-time employment	36,439	0.668	0.471	0	1	1
Have basic old-age insurance	36,439	0.651	0.477	0	1	1
**Retirement support**						
Relying on children for retirement	36,439	0.655	0.475	0	1	1
Relying on savings for retirement	36,439	0.0390	0.194	0	0	1
Relying on pension or retirement salary for retirement	36,439	0.254	0.435	0	0	1
Relying on commercial pension insurance for retirement	36,439	0.00400	0.0600	0	0	1
Relying on other for retirement	36,439	0.0480	0.215	0	0	1
Have UEBMI	36,439	0.0990	0.298	0	0	1
Have URBMI	36,439	0.0430	0.203	0	0	1
Have URRBMI	36,439	0.0370	0.190	0	0	1
Hospitalization	36,439	0.129	0.335	0	0	1
Log (inpatient out-of-pocket expenses + 1)	36,439	0.293	1.545	0	0	11.98
**Household-level covariates**						
Log (household debt + 1)	36,439	1.421	3.523	0	0	18.76
Number of household members	36,439	3.502	1.847	1	3	16
Number of household durables	36,439	4.603	2.184	0	5	18
Household education andand training expenses	36,439	1.344	2.985	0	0	12.76

## 4. Analytical strategies

To assess the impact of LTCI on multidimensional poverty among middle-aged and older adults, we applied a DID strategy controlling for individual fixed effects (FE) to panel data from 2011 to 2018. The treatment group in this study was middle-aged and older adults in the pilot city who were covered by LTCI, while the control group was middle-aged and older adults who were not covered by LTCI. The following equation is estimated:


(6) $$\begin{array}{l} y_{c_{i}t}=\alpha_{1}LTCI_{c_{i}t}+\alpha_{2}X_{c_{i}t}+\alpha_{3}w_{c_{h}t}+\sigma_{t}+\tau_{i}+\varepsilon_{c_{i}t} \end{array}$$

where *y*_*c*_*i*_*t*_ denotes the outcome variable for individual *i* living in the city *c* in year *t*, including the IMD and indicators of income poverty, living consumption poverty, material wellbeing poverty, social participation poverty, health poverty, and psychological wellbeing poverty derived from the decomposition of the IMD by dimension.

The key independent variable *LTCI*_*c*_*i*_*t*_ has two set forms. One form is *Treat*_*c*_*i*__×*Post*_*t*_, where *Post*_*t*_ is a dichotomous variable that is 0 before city *c* starts the LTCI pilot and 1 after it starts the LTCI pilot, and *Treat*_*c*_*i*__ is a dummy variable for individual treatment status, defined according to the LTCI coverage group and individual basic health insurance status in the pilot city. τ_*i*_ explains all time-invariant factors that may affect the outcome variables. Another form of *LTCI*_*c*_*i*_*t*_ is the duration of LTCI coverage, which is defined by the specific time of implementation of the LTCI program in each pilot city and the date of interview of the surveyed individuals.

*X*_*c*_*i*_*t*_ is a vector of individual time-varying characteristics associated with demographic and socioeconomic characteristics, and *w*_*c*_*h*_*t*_ is a set of household-by-year controls, as described in Table 2A. σ_*t*_ is a year-fixed effect and ε_*c*_*i*_*t*_ is a random error term. Standard errors are clustered at the city level to account for possible correlations between middle-aged and older adults in the same city.

Because our data set does not allow us to directly observe whether individuals are served by LTCI, the coefficient α_1_ is an estimate of the net effect of expanding LTCI coverage, averaged across covered middle-aged and older adults who do or do not yet receive LTCI services.

The issue of concern is the problem of sample selection bias. To avoid sample selection bias, we borrowed an idea from Imbens and Wooldridge (49) and combined the propensity score matching (PSM) method with DID regression. This method is also known as PSM-DID. First, we calculated propensity scores using a logit model (i.e., to estimate the probability of entering the treatment group given a set of observable characteristics including demographic and socioeconomic covariates, and household-level covariates). Second, based on the estimated propensity scores, we estimated DID for individuals who met the common support hypothesis, and individuals who did not meet the common support hypothesis were excluded from the DID analysis. We used a matching strategy of year-by-year matching as well as six nearest neighbor matching. All observable features were better balanced after matching, and the propensity score distributions were more similar for both groups (see Appendix Tables D4, D5; Appendix Figure D4).

Another issue of concern to us is the small sample size of the treatment group. Theoretically, a smaller proportion of treatment groups relative to control groups does not pose a significant problem for estimation in the data structure of this paper, and DID estimation does not require that the treatment and control groups' relative proportions meet a specific criterion. The only purpose of the control group is to provide a counterfactual for the treatment group. The sample size of the treatment group in Moser and Voena (50)'s classic study is only 4.6%. In the robustness test, we attempt to design a test procedure and use the synthetic difference-differences (SDID) method to verify that the small sample proportion of the treatment group does not affect the robustness of the paper's findings.

## 5. Results

### 5.1. Main effects of LTCI

Table 3 reports the estimated impact of LTCI coverage on IMD. Each column in the table corresponds to the results of a separate regression. The first column shows that the LTCI coverage significantly reduces multidimensional poverty. In column 2, the DID estimates vary slightly in magnitude based on samples that meet the common support assumption, but the direction remains the same as in column 1. The coefficient of the interaction term of Treat and Post is −0.09 is statistically significant at the level of 0.01, which indicates that IMD decreased 21.53% after the implementation of LTCI. In Columns 3 and 4, we also study the effect of LTCI coverage duration. Column 3 shows that LTCI duration has a significant negative effect on IMD. In column 4, based on samples that meet the common support assumption, we find that the coefficient of LTCI duration is −0.044 is statistically significant at the level of 0.01, which indicates that IMD decreased 10.53% for one more year of LTCI coverage.

**Table 3:** Effect of LTCI on IMD.

	**Coefficient on treat** × **post**		**Coefficient on LTCI_duration**	
	**DID**	**DID with matching**	**DID**	**DID with matching**
	**(1)**	**(2)**	**(3)**	**(4)**
Treat × post	−0.064^***^ (0.023)	−0.090^***^ (0.034)		
LTCI_duration			−0.032^***^ (0.012)	−0.044^***^ (0.015)
Age	−0.000 (0.001)	0.004 (0.003)	−0.000 (0.001)	0.004 (0.003)
Gender	0.022 (0.022)	−0.101 (0.063)	0.022 (0.022)	−0.101 (0.063)
Marital status	0.014^*^ (0.008)	−0.004 (0.025)	0.014^*^ (0.008)	−0.004 (0.025)
Hukou status	−0.015 (0.022)	−0.035 (0.046)	−0.014 (0.022)	−0.033 (0.046)
Level of education [Reference group: No formal education (illiterate)]			
Did not finish primary school but capable of reading or writing	−0.012 (0.008)	−0.022 (0.018)	−0.012 (0.008)	−0.022 (0.018)
Sishu/home school	−0.071^**^ (0.029)	—	−0.071^**^ (0.029)	—
Graduate from elementary school	−0.024^***^ (0.009)	−0.051^**^ (0.020)	−0.024^***^ (0.009)	−0.051^**^ (0.020)
Graduate from middle school	−0.022^*^ (0.012)	−0.043^*^ (0.026)	−0.022^*^ (0.012)	−0.044^*^ (0.026)
Graduate from high school	0.008 (0.019)	0.001 (0.032)	0.007 (0.019)	0.000 (0.032)
Graduate from vocational school	−0.002 (0.022)	−0.037 (0.039)	−0.002 (0.022)	−0.034 (0.039)
Graduate from 2/3 year college/associate degree	0.005 (0.024)	−0.131^**^ (0.059)	0.005 (0.024)	−0.127^**^ (0.059)
Graduate from 4 year college/bachelor's degree	−0.040 (0.050)	—	−0.040 (0.050)	—
Full-time employment	−0.005 (0.003)	0.006 (0.007)	−0.005 (0.003)	0.006 (0.007)
Have basic old-age insurance	−0.015^***^ (0.003)	−0.006 (0.009)	−0.015^***^ (0.003)	−0.006 (0.009)
Retirement support (Reference group: Relying on children for retirement)			
Relying on savings for retirement	−0.003 (0.006)	−0.005 (0.028)	−0.003 (0.006)	−0.005 (0.028)
Relying on pension or retirement salary for retirement	0.003 (0.004)	0.017^*^ (0.009)	0.003 (0.004)	0.017^**^ (0.009)
Relying on commercial pension insurance for retirement	0.020 (0.016)	—	0.020 (0.016)	—
Relying on others for retirement	0.023^***^ (0.005)	0.022 (0.018)	0.023^***^ (0.005)	0.022 (0.018)
Have UEBMI	−0.012^*^ (0.006)	−0.007 (0.017)	−0.012^*^ (0.006)	−0.009 (0.017)
Have URBMI	0.001 (0.007)	0.060^***^ (0.019)	0.001 (0.007)	0.060^***^ (0.019)
Have URRBMI	0.002 (0.008)	0.025^**^ (0.011)	0.002 (0.008)	0.025^**^ (0.011)
Hospitalization	−0.001 (0.003)	−0.006 (0.008)	−0.001 (0.003)	−0.007 (0.008)
Log (Inpatient out-of-pocket expenses + 1)	0.001 (0.001)	0.004 (0.003)	0.001 (0.001)	0.004 (0.003)
Log (Household debt + 1)	0.000 (0.000)	0.002^*^ (0.001)	0.000 (0.000)	0.002^*^ (0.001)
Number of household members	0.005^***^ (0.001)	0.006^*^ (0.003)	0.005^***^ (0.001)	0.006^*^ (0.003)
Number of household durables	−0.008^***^ (0.001)	−0.002 (0.002)	−0.008^***^ (0.001)	−0.002 (0.002)
Household education and training expenses	−0.005^***^ (0.000)	−0.005^***^ (0.001)	−0.005^***^ (0.000)	−0.005^***^ (0.001)
_cons	0.459^***^ (0.052)	0.288 (0.183)	0.459^***^ (0.052)	0.288 (0.183)
Observations	36,439	11,896	36,439	11,896
Adj-*R*^2^	0.320	0.274	0.320	0.274
Individual FE	Y	Y	Y	Y
Year FE	Y	Y	Y	Y

Robust standard errors are clustered at the city level. ^***^, ^**^, and ^*^ denote 1, 5, and 10% significance level, respectively. All regressions control for year FE, individual FE, demographic and socioeconomic covariates, and household-level covariates, and the specific covariates controlled for are detailed in Table 2A.

We further analyzed the effect of LTCI on each dimension of poverty. The regression results in Column 2 of Table 4 are based on samples that meet the common support assumption that LTCI coverage is associated with a 27.7% reduction in the likelihood of income poverty and a 12.2% reduction in the likelihood of health poverty. For living consumption poverty, the estimate is negative and significant in the first column (*p* = 0.041), but statistically insignificant in the second column. Column 4 in Table 4 estimates the effect of LTCI coverage time on each dimension of poverty based on the sample that meets the common support assumption. According to the findings, one more year of LTCI coverage reduces the likelihood of income poverty by 11.9% and subsistence consumption poverty by 9.6%. This implies that as the duration of LTCI coverage increases, it will have a poverty-reducing effect on the living consumption dimension. In contrast, the estimates for health poverty are not statistically significant, indicating that LTCI coverage does not reduce poverty in the health dimension over time.

**Table 4:** Effect of LTCI on poverty in each dimension.

	**Coefficient on treat** × **post**		**Coefficient on LTCI duration**	
	**DID**	**DID with matching**	**DID**	**DID with matching**
	**(1)**	**(2)**	**(3)**	**(4)**
Income poverty	−0.107^***^ (0.038)	−0.277^***^ (0.079)	−0.055^***^ (0.019)	−0.119^***^ (0.036)
Observations	36,439	11,896	36,439	11,896
Adj-*R*^2^	0.291	0.256	0.291	0.256
Living consumption poverty	−0.167^**^ (0.081)	−0.129 (0.120)	−0.098^***^ (0.035)	−0.096^**^ (0.041)
Observations	36,439	11,896	36,439	11,896
Adj-*R*^2^	0.261	0.231	0.261	0.232
Material wellbeing poverty	−0.066 (0.043)	−0.026 (0.033)	−0.022 (0.016)	−0.015 (0.024)
Observations	36,439	11,896	36,439	11,896
Adj-*R*^2^	0.363	0.408	0.363	0.408
Social participation poverty	−0.047 (0.052)	−0.023 (0.061)	−0.020 (0.026)	−0.023 (0.030)
Observations	36,439	11,896	36,439	11,896
Adj-*R*^2^	0.228	0.268	0.228	0.269
Health poverty	−0.066 (0.050)	−0.122^*^ (0.066)	−0.029 (0.026)	−0.026 (0.040)
Observations	36,439	11,896	36,439	11,896
Adj-*R*^2^	0.215	0.096	0.215	0.095
Psychological wellbeing poverty	−0.000 (0.037)	−0.010 (0.061)	0.006 (0.023)	0.014 (0.041)
Observations	36,439	11,896	36,439	11,896
Adj-*R*^2^	0.299	0.303	0.299	0.303
Individual FE	Y	Y	Y	Y
Year FE	Y	Y	Y	Y

Robust standard errors are clustered at the city level. ^***^, ^**^, and ^*^ denote 1, 5, and 10% significance level, respectively. All regressions control for year FE, individual FE, demographic and socioeconomic covariates, and household-level covariates, as specified in Table 3.

Middle-aged and older adults who already have LTCI coverage are only eligible for LTC services if they have an ADL hardship condition. Although CHARLS does not have direct information on individuals' service status for the LTCI program, we added an interaction term in Table 5 between LTCI coverage and whether the individual surveyed had ADL difficulties in 11 areas (i.e., dressing, bathing, eating, getting in and out of bed, going to the bathroom, controlling bowel movements, doing chores, cooking, shopping, managing money, and taking medication). We examined the difference in the LTCI poverty reduction effect between subgroups with and without LTC demand in the group already covered by LTCI using this interaction term. In our dataset, 14.98% of the population had ADL difficulties. The coefficient of the interaction term can be interpreted as the differential effect of LTCI on middle-aged and older adults with LTC needs relative to those without LTC needs.

**Table 5:** Effect of LTCI with LTC need and without LTC need.

**Dependent variables**	**DID with matching**			
	**(1)**		**(2)**	
	**Coefficient on treat × post × no LTC need**	**Coefficient on treat × post × have LTC need**	**Coefficient on LTCI duration × no LTC need**	**Coefficient on LTCI duration × have LTC need**
IMD	−0.083^***^ (0.031)	−0.158^***^ (0.059)	−0.043^***^ (0.014)	−0.052^*^ (0.028)
Income poverty	−0.275^***^ (0.094)	−0.294^*^ (0.158)	−0.124^**^ (0.050)	−0.091 (0.076)
Living consumption poverty	−0.099 (0.112)	−0.402^***^ (0.144)	−0.084^**^ (0.040)	−0.153^***^ (0.056)
Material wellbeing poverty	−0.021 (0.044)	−0.074 (0.233)	−0.011 (0.017)	−0.031 (0.128)
Social participation poverty	−0.018 (0.063)	−0.072 (0.054)	−0.022 (0.034)	−0.032^*^ (0.017)
Health poverty	−0.124^*^ (0.072)	−0.100^**^ (0.045)	−0.026 (0.047)	−0.026^*^ (0.014)
Psychological wellbeing poverty	−0.008 (0.054)	−0.033 (0.226)	0.014 (0.035)	0.017 (0.102)

Robust standard errors are clustered at the city level. ^***^, ^**^, and ^*^ denote 1, 5, and 10% significance level, respectively. All regressions control for year FE, individual FE, demographic and socioeconomic covariates, and household-level covariates, as specified in Table 3.

The first two columns in each row of Table 5 indicate the interaction DID estimates of LTCI coverage without and with LTC need, respectively. The last two columns show the interaction DID estimates of LTCI duration without and with LTC need. For multidimensional poverty status, the effect of LTCI on improving the multidimensional poverty status of middle-aged and elderly people with LTC needs is significantly greater than that of middle-aged and elderly people without LTC needs, and the covered population with LTC needs may be the actual recipients of LTCI benefits. For middle-aged and older adults with LTC needs, the effect of LTCI coverage on the reduced likelihood of income poverty occurrence is significantly stronger than for those without LTC needs. In the long run, LTCI would reduce the likelihood of income poverty by 12.4% for middle-aged and older adults without LTC needs for one more year of LTCI coverage, while the estimates for middle-aged and older adults with LTC needs were not statistically significant. This phenomenon may be attributed to the fact that LTCI coverage may have a “peace of mind” effect on middle-aged older adults even if they do not receive benefits (51), or that these middle-aged and older adults without LTC needs are more likely to be informal caregivers who benefit from LTCI's reduced informal caregiving burden (29).

For living consumption poverty, LTCI coverage only reduced the likelihood of living consumption poverty by 40.2% for middle-aged and older adults with LTC needs, while the estimate for middle-aged and older adults without LTC needs was not statistically significant. Estimates for the duration of LTCI coverage showed that one more year of LTCI coverage had a significantly greater effect on reducing the likelihood of living consumption poverty for middle-aged and older adults with LTC needs than for middle-aged and older adults without LTC needs. For social participation poverty, LTCI coverage only reduces the likelihood of social participation poverty among middle-aged and older adults with LTC needs in the long run, with one more year of LTCI coverage being associated with a 3.2% reduction in the likelihood of social participation poverty among middle-aged and older adults with LTC needs.

For health poverty, coverage of LTCI has a significantly greater effect on reducing the likelihood of health poverty occurring in the middle-aged and elderly population without LTC needs than in the middle-aged and elderly population with LTC needs. One possible explanation is that in addition to the “peace of mind” effect of LTCI coverage and the fact that these middle-aged and elderly people without LTC needs may be informal caregivers, the reduced likelihood of health poverty due to the reduced caregiving burden, the rich community services provided by the LTCI pilot (e.g., regular medical checkups, home visits, etc.) reduce the likelihood of health poverty among middle-aged and older adults without LTC needs. For middle-aged and elderly people with LTC needs, their health status may not be good for a long time, and they may have chronic diseases that are difficult to cure, so the effect of basic LTCI services on the health of this group may be very limited. In the long term, one more year of LTCI coverage will reduce the likelihood of health poverty among the middle-aged and elderly population with LTC needs by 2.6%.

In the sensitivity analysis, we used methods such as converting unbalanced panels to balanced panels, replacing the outcome variables using logit models, converting time-varying DID estimates to ordinary DID estimates, and using the second batch of LTCI pilot cities as the control group. Furthermore, we conducted a sensitivity analysis for different deprivation dimension thresholds k and discovered that different k does not affect the results of this paper. The simplicity of the IMD construction makes it flawed. The IMD assumes that the dimensions are independent of one another, but they are not. For example, the material wellbeing dimension influences the health dimension. The IMD cannot more precisely target a specific poor group, it does not change when a good is transferred from poor to non-poor households. As a result, IMD cannot more precisely identify individuals who are closest to escaping poverty or who are most in need of assistance. We use the correlation-sensitive poverty index (CSPI) constructed by Rippin (52), also known as the multidimensional inequality index, to conduct a sensitivity analysis that addresses the shortcomings of the IMD. The unique structure of the CSPI allows it to overcome the shortcomings of the IMD. The CSPI does not require that the dimensions be independent of each other and can identify the neediest groups. We found similar results (see Appendix C).

### 5.2. Robustness tests

The DID estimation premise assumes that when the policy is not implemented, the trends of change in the treatment and control groups should be parallel. We use the event study method to see if the parallel trend assumption holds, and we find no evidence of heterogeneity in the trends of change between the treatment and control groups before treatment (see Appendix Figure D1). Furthermore, to strengthen the credibility of the parallel trend hypothesis, we used Abadie SDID reweighting regression for validation. The Abadie SDID reweighting regression is a reweighting technique that can be used to deal with the imbalance of characteristics between the treatment and control groups, which can make the conclusion somewhat plausible even if the parallel trend hypothesis is not fully satisfied (53). Thus, the technique increases the credibility of the parallel trend hypothesis. The results of the Abadie SDID reweighted regression make the parallel trend hypothesis of this paper more plausible (see Appendix Table D1).

Furthermore, We devised a test procedure to ensure that the robustness of the findings in this paper is not compromised by the treatment group's small proportion of the sample size. We took 10% of the samples from the control group each time as a new control group, combined with the treatment group to perform a regression according to Equation (6), repeated this regression 1,000 times, and plotted the density distribution of the regression coefficient α_1_ for these 1,000 regressions. We find that the baseline regression coefficients are very close to the median of the density function, confirming that the conclusions of this paper are not affected by the small proportion of sample size in the treatment group (see Appendix Figure D2).

Further, we use the synthetic difference-differences (SDID) method to verify that the small sample proportion of the treatment group does not affect the robustness of the paper's findings. Usually, we can use the synthetic control method (SCM) to assess policy treatment effects when the treatment group contains only one individual or very few individuals. Arkhangelsky et al. (54) combine the SCM with DID to form the SDID, which can find control group individuals similar to the treatment group by individual weights and also find the post-policy treatment period by time weights and assign them larger individual and time weights, respectively. The SDID estimation results show that the small proportion of the sample in the treatment group does not affect the robustness of the findings in this paper (see Appendix Table D2).

Another concern we have is that the LTCI pilot cities may not be chosen at random. The central or local government decides on LTCI implementation, which may be influenced by some unique characteristics of the city chosen to conduct the LTCI pilot. To alleviate this problem, we control for all observable and unobservable heterogeneity at the city level that does not change over time by controlling for city-level fixed effects, which have been absorbed by individual fixed effects. We further incorporate city-year interactions to control for the effects of urban characteristics over time and still find the conclusions of this paper to be robust (see Appendix Table D3). Furthermore, we used a placebo test to ensure that this possible nonrandomization has no negative impact on the robustness of the findings in this paper. We conducted a placebo test by randomly selecting several cities as pseudo-treatment group cities. The results of the placebo test suggest that this possible non-randomness in the selection process of LTCI pilot cities does not have a deleterious effect on the robustness of our study findings (see Appendix Figure D3).

Goodman-Bacon et al. (55) show that the time-varying DID estimates based on the occurrence of policies at multiple points in time consist of four 2x2 DID estimates, and the final policy treatment effects are obtained by weighting these four 2 × 2 DID estimators. The new treatment group may take the previous treatment group as the control group, which we call the “bad control group.” We are concerned that such “bad control groups” may also exist in our time-varying DID estimation, thus biasing the estimation results in this paper. We decompose the time-varying DID estimates in this paper using the Bacon decomposition to verify that the possible presence of the “bad control group” does not affect the robustness of the conclusions in this paper (see Appendix Table D6 and Appendix Figure D5). The results of the Bacon decomposition suggest that the possible presence of the “bad control group” in our DID estimation does not affect the robustness of the findings in this paper.

### 5.3. Effect of LTCI on multidimensional poverty vulnerability

We continue to use the estimation strategy of Equation (6) to evaluate the impact of LTCI on MPV. We first tested the hypothesis that the treatment and control groups had similar time trends in MPV before treatment using an event study approach, and the event study analysis results for MPV measured at *k* = 1/3 and *k* = 2/3, respectively, are detailed in Appendix Figures E1, E2. Whether *k* = 1/3 or *k* = 2/3, the pre-reform estimates were all non-significant. This indicates that the trend of MPV in the treatment group does not differ from that of the control group in the pre-reform period. In contrast, MPV declined significantly in the reform year and even more in the year following the reform year. This finding provides additional evidence that the trend of this effect remains consistent with the true effect.

In Table 6, we estimated the MPV for regression by taking 1/3 and 2/3 for *k* (deprivation dimension threshold), respectively. LTCI coverage/duration had a significant negative effect on MPV whether *k* was taken as 1/3 or 2/3, implying that LTCI can significantly reduce an individual's likelihood of future multidimensional poverty. The effect of LTCI coverage/duration on MPV was significantly greater in the middle-aged and elderly with LTC needs than in the middle-aged and elderly without LTC needs. Using *k* = 1/3, for those middle-aged and older adults with LTC needs, LTCI coverage was associated with a 20.5−38.7 reduction in MPV; for those middle-aged and older adults without LTC needs, LTCI coverage was associated with a 13%−19.3% reduction in MPV.

**Table 6:** Effect of LTCI on MPV.

	**MPV (*****k*** = **1/3)**				**MPV (*****k*** = **2/3)**			
	**DID**		**DID with matching**		**DID**		**DID with matching**	
	**(1)**	**(2)**	**(3)**	**(4)**	**(5)**	**(6)**	**(7)**	**(8)**
**Panel A**								
Treat × post	−0.138^**^ (0.059)		−0.211^**^ (0.086)		−0.009^***^ (0.002)		−0.010^***^ (0.002)	
Treat × post × no LTC need		−0.130^**^ (0.057)		−0.193^**^ (0.079)		−0.008^***^ (0.002)		−0.009^***^ (0.002)
Treat × post × have LTC need		−0.205^**^ (0.093)		−0.387^***^ (0.131)		−0.073^***^ (0.017)		−0.021^**^ (0.009)
**Panel B**								
Treat duration	−0.076^***^ (0.029)		−0.101^***^ (0.035)		−0.005^***^ (0.001)		−0.005^***^ (0.001)	
Treat duration × no LTC need		−0.073^**^ (0.028)		−0.101^***^ (0.035)		−0.005^***^ (0.001)		−0.005^***^ (0.001)
Treat duration × have LTC need		−0.091^**^ (0.036)		−0.137^**^ (0.052)		−0.007^***^ (0.002)		−0.007^**^ (0.003)
Observations	36,439	36,439	11,896	11,896	36,439	36,439	11,896	11,896
Adj-*R*^2^	0.741	0.741	0.643	0.643	0.414	0.414	0.474	0.958
Year FE	Y	Y	Y	Y	Y	Y	Y	Y
Individual FE	Y	Y	Y	Y	Y	Y	Y	Y

Robust standard errors are clustered at the city level. ^***^, ^**^, and ^*^ denote 1, 5, and 10% significance level, respectively. All regressions control for year FE, individual FE, demographic and socioeconomic covariates, and household-level covariates, as specified in Table 3.

### 5.4. Heterogeneity

**Figure 3:**
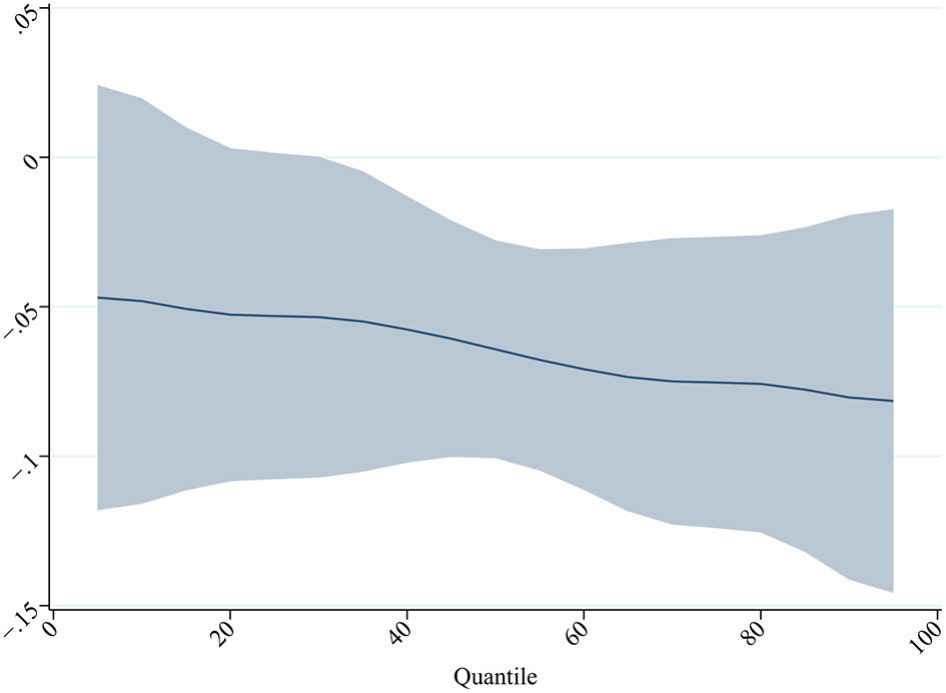
Effect of LTCI on individuals in different quartiles of MPI. The figure depicts the coefficients as well as the 90% confidence intervals for the regression analysis. Robust standard errors are clustered at the city level. ^***^, ^**^, and ^*^ denote 1%, 5%, and 10% significance level, respectively. All regressions control for year FE, individual FE, demographic and socioeconomic covariates, and household-level covariates, as specified in Table 3.

First, we are interested in the heterogeneity of the impact of LTCI on individuals at different quartiles of the IMD. The greater the multidimensional poverty of an individual, the more difficult it is to reduce poverty. If LTCI has a greater effect on poverty reduction for individuals with more severe multidimensional poverty, this implies that LTCI plays a “timely help” role in poverty reduction. We estimated the effect of LTCI on individuals in different quartiles of IMD using quantile regression. The results are shown in Figure 3 and Appendix Table F1.

We find that the quantile estimates for both specifications suggest that LTCI has a greater impact on middle-aged and older adults in the higher quantile of multidimensional poverty. Not only does LTCI significantly reduce multidimensional poverty among middle-aged and older adults, but it also plays a greater role for the group of middle-aged and older adults who need the most help. One possible explanation is that, on the one hand, LTCI in China provides different levels of benefit based on the level of disability, and the more severe the disability, the better the LTCI benefit, providing for a reduction in multidimensional poverty for individuals with severe multidimensional poverty who may have received LTCI services. On the other hand, individuals who are covered by LTCI but do not receive LTCI services may be informal caregivers of individuals who have received LTCI services and benefit from the reduction in multidimensional poverty of these individuals who have received LTCI services.

**Figure 4:**
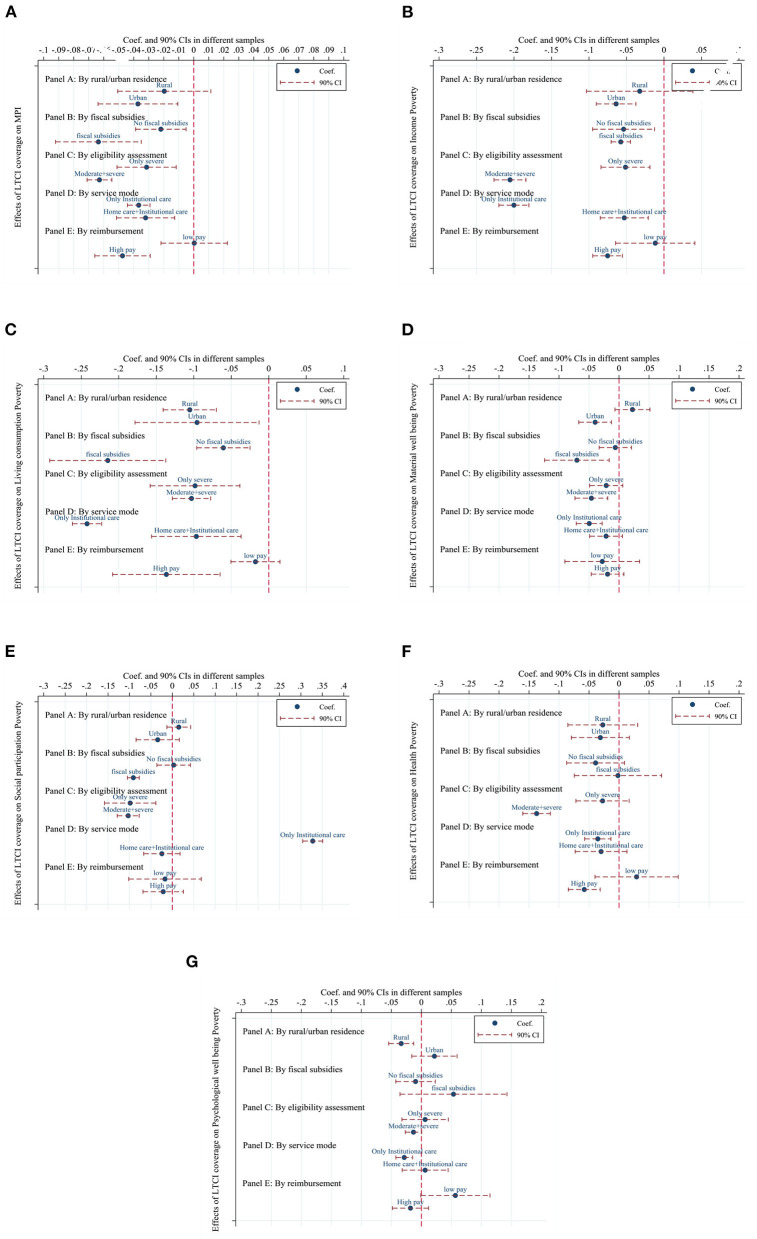
Heterogeneous effects of LTCI. The figure depicts the coefficients as well as the 90% confidence intervals for the regression analysis. Robust standard errors are clustered at the city level. ^***^, ^**^, and ^*^ denote 1%, 5%, and 10% significance level, respectively. All regressions control for year FE, individual FE, demographic and socioeconomic covariates, and household-level covariates, as specified in Table 3. **(A)**, IMD; **(B)**, Income poverty; **(C)**, Living consumption poverty; **(D)**, Material wellbeing poverty; **(E)**, Social participation poverty; **(F)**, Health poverty; **(G)**, Psychological wellbeing poverty.

Another interesting question worth investigating is whether the relationship between LTCI and each outcome variable may differ in some dimensions. We interact LTCI duration with the indicators of each subgroup, and the estimated coefficients of each interaction term show the estimated effect of the subgroup with certain observable characteristics. The results are shown in Figure 4 and Appendix Table F2 in the Appendix. The dotted lines in Figure 4 represent the estimated coefficients of the effect of LTCI duration on each subgroup outcome variable, and the dashed lines represent the 90% confidence intervals for each estimate.

In Figure 4A, each graph shows the estimated results for each outcome variable by rural/urban residents. For the rural resident group, LTCI coverage has a significant negative effect on IMD, the likelihood of income poverty, and the likelihood of material wellbeing poverty for urban residents. In the living consumption poverty dimension, LTCI coverage has a significant negative effect on both rural residents and urban residents, but the effect on rural residents is greater than that on urban residents. Furthermore, LTCI coverage only has a significant negative effect on the likelihood of psychological wellbeing poverty for rural residents, and the effect on urban residents is not statistically significant.

In Figure 4B, each graph shows the estimated results for each outcome variable by fiscal subsidies. For the fiscal subsidy group, LTCI coverage has a significant negative effect on IMD, the likelihood of income poverty, the likelihood of living consumption poverty, the likelihood of social participation poverty, and the likelihood of material wellbeing poverty for individuals in pilot cities with fiscal subsidies. Except for the IMD, income poverty and living consumption poverty dimensions, the estimates for the no-subsidy group are not significant in other dimensions. In the income poverty dimension and the living consumption poverty dimension, the LTCI coverage has a significantly larger effect on the group with fiscal subsidies relative to the group without fiscal subsidies.

In Figure 4C, each graph shows the estimated results for each outcome variable by eligibility assessment. The negative effects of LTCI coverage on IMD, the likelihood of income poverty, the likelihood of living consumption poverty, and the likelihood of social participation poverty were significantly greater for the group with moderately disabled and severely disabled people as enrollees than for the group with severely disabled people only as enrollees. In the material welfare poverty and health poverty dimensions, LTCI coverage had statistically insignificant estimates for the group with only the severely disabled as enrollees, while it had a statistically significant negative effect for the group with the moderately disabled and severely disabled as enrollees.

In Figure 4D, each graph shows the estimated results for each outcome variable by service mode. The negative effect of LTCI coverage on IMD, the likelihood of income poverty, and the likelihood of living consumption poverty was significantly greater for the group with institutional care only as the service model than for the group with home care and institutional care as the service model. In the material wellbeing poverty, health poverty, and psychological wellbeing poverty dimensions, the LTCI coverage was not statistically significant in its estimates for the group with home care and institutional care as a service model, while it had a significant negative effect on the group with institutional care only as a service model. In the social participation poverty dimension, LTCI coverage significantly increased the likelihood of social participation poverty occurring in the group with institutional care only as a service model.

In Figure 4E, each graph shows the estimated results for each outcome variable by reimbursement. Considering LTCI reimbursement rates above 75% and daily payments above 100 RMB as high pay, the pilot cities were divided into high and low-pay groups. The estimates of LTCI coverage on IMD for the low-pay group were not statistically significant, while it had a significant negative effect on IMD for the high-pay group. In the income poverty, living consumption poverty, and health poverty dimensions, the estimates of LTCI coverage were not statistically significant for the low-pay group, while it had a significant negative effect on the high-pay group.

## 6. Discussion and conclusion

As the population ages and the family structure becomes smaller, the function of families to provide informal care services weaken, while demand for formal LTC services rise in China. To explore the feasibility of establishing an LTCI system, the central government launched the LTCI pilot program in 2016. Using longitudinal data based on a national random sample survey, this paper used a DID strategy to assess the effect of LTCI coverage in improving multidimensional poverty status and unidimensional poverty status and in reducing multidimensional poverty vulnerability. As one of the few studies evaluating the impact of the LTCI pilot, this paper provides evidence that the implementation of LTCI reduced multidimensional poverty and multidimensional poverty vulnerability among middle-aged and older adults.

We found that the IMD of middle-aged and older adults decreased significantly 21.53% after the implementation of LTCI and that one more year of LTCI coverage was associated with a significant 10.53% decrease in IMD. LTCI had a greater impact on middle-aged and older adults with higher quartiles of multidimensional poverty. Furthermore, we found that LTCI coverage contributed to a reduction in MPV and that the impact was greater for individuals with LTC needs. These findings provide evidence for the existence of LTCI poverty reduction effects and confirm that LTCI coverage improves multidimensional poverty among covered individuals and reduces their likelihood of future multidimensional poverty.

For income poverty and living consumption poverty, we found that LTCI coverage significantly reduces the likelihood of income poverty among the middle-aged and elderly by 27.7%, and LTCI coverage significantly reduces the likelihood of living consumption poverty among individuals with LTC needs by 40.2%. These effects may stem from the reduction in care and medical expenditures due to LTCI and the increase in the labor supply of informal family caregivers. The literature suggests that public LTCI programs in China are associated with reductions in out-of-pocket medical costs. Lei et al. (29) found a 23.5% reduction in out-of-pocket medical costs for covered individuals for one more year of LTCI coverage. Lu et al. (19) and Feng et al. (20) similarly found this phenomenon in their studies of LTCI pilots in Shanghai and Qingdao, China. The implementation of public LTCI programs in China has also reduced the caregiving burden of family caregivers, and Lei et al. (29) found that LTCI coverage resulted in a 47% reduction in informal caregiving time. The reduced burden of informal caregiving implies a potential positive spillover effect of LTCI on the labor supply of family caregivers (22). The public LTCI program in China also provides cash subsidies to covered individuals who choose family caregivers for home care.

We also found some evidence that LTCI can improve health poverty and social participation poverty. Coverage of LTCI significantly reduces the likelihood of health poverty by 12.2%, and one more year of LTCI coverage significantly reduces the likelihood of health poverty for individuals with LTC needs by 2.6%. These findings are similar to those of Stabile et al. (27), who found a positive effect of publicly funded home care services in Canada on the self-rated health of welfare recipients. Physical function decline in older adults who are physically frail can be slowed by a randomized, family-based intervention program (56). However, some studies have shown that more LTC services do not have an impact on health outcomes (57). One possible explanation is that China has a high demand for LTC but a chronic lack of LTC services, so LTCI coverage has an impact on health poverty. Additionally, our findings can be compared with those of Lei et al. (29), who discovered that public LTCI programs in China resulted in a 29% increase in the likelihood of self-rated good health status among individuals in need of care. In the social participation poverty dimension, we found that one more year of LTCI coverage reduced the likelihood of occurrence of social participation poverty by 3.2% for individuals with LTC needs. This may be related to improvements in the health status of covered individuals and the care services provided by LTCI.

Furthermore, we focused on the effect of LTCI coverage on different subgroups. LTCI coverage has a much greater reduction effect on the likelihood of living consumption poverty for rural residents than for urban residents, and a statistically significant reduction effect on the likelihood of psychological wellbeing poverty for rural residents only, while it is statistically insignificant for urban residents. However, LTCI coverage only affects IMD, the likelihood of income poverty, and the likelihood of material wellbeing poverty for urban residents, and is not significant for rural residents. These findings may be related to the fact that the LTCI pilot began supplying care services to rural residents, but care resources for rural residents are still undersupplied relative to urban residents (58). We also found that the impact of LTCI coverage on IMD, the likelihood of income poverty, and the likelihood of living consumption poverty was significantly greater in pilot cities that offered only one service model, institutional care, than in pilot cities that offered both institutional care and home care. One possible explanation is that older adults in China prefer home care to institutional care (59), so the burden of informal caregivers in pilot cities that only provide institutional care is lower than in pilot cities that provide both home and institutional care. This explanation also applies to estimates in the material wellbeing poverty dimension. Estimates on the likelihood of health and psychological wellbeing poverty indicate that LTCI coverage has a significant impact only on individuals in pilot cities that only provide institutional care. This could be because institutional care is more specialized in terms of care circumstances, care equipment, and caregivers than home care. However, we found that LTCI coverage in LTCI pilot cities that provided only institutional care significantly increased the likelihood of socially engaged poverty occurrence. This finding may be related to barriers to social participation among individuals requiring LTC services in institutional care settings. Studies of older residents requiring LTC services in institutional care settings in China have confirmed the existence of this barrier to social participation (60).

Additionally, we found that the impact was greater for LTCI programs that provided fiscal subsidies, included both severe and moderate disability in the granting of benefits, and higher benefit payments. It is important to optimize the existing LTCI system design by assessing the heterogeneity of the effects of LTCI design in different pilot cities to help establish a suitable public LTCI system in China.

There are several limitations in our study. First, the literature suggests that public programs, such as cash transfers, can indirectly affect ineligible individuals in the same area (61). Because specific information on individual access to LTCI benefits is not available, the treatment effects estimated in this paper include both direct effects for direct LTCI beneficiaries and spillover effects for non-direct LTCI beneficiaries who are covered by LTCI programs. Therefore, this paper may have underestimated the impact of the LTCI program on actual beneficiaries. Second, due to data limitations, we do not know which service model was selected by individuals who received LTCI benefits, so the impact of home care, institutional care, and hospital care cannot be estimated separately in this paper. Overall, this study provides empirical evidence for the effectiveness of LTCI in improving multidimensional poverty among middle-aged and elderly people in China. These findings have important policy implications for further optimizing LTCI program design and expanding LTCI pilots in China, as well as for developing LTCI systems in other developing countries with rapidly growing disabling populations and LTC needs.

## Author's note

Disability is one of the major causes of poverty, people with disabilities and their families are more likely to fall into poverty than families without a disabled person. However, the vast majority of care services are provided and paid for by families and individuals, only 6% of the world's population has access to government-sponsored long-term care assistance. When the price of care is solely determined by the market, individuals in need of care can quickly fall into a poverty trap due to costly care. The traditional model of informal care provided by families in China is unsustainable as the proportion of nuclear families rises. Family members are tethered to provide care for relatives with disabilities, making it difficult for them to find work and miss out on development opportunities, leaving families in distress. However, poverty does not only mean low income, but also a lack of capability. Therefore, this paper examines the poverty reduction effects of the public long-term care insurance (LTCI) program piloted in China in recent years from a multidimensional poverty perspective. The findings of this paper suggest that the establishment of an LTCI system can improve the poverty of middle-aged and older adults in several ways, which has important implications for the development of LTCI systems in China and other developing countries.

## Data availability statement

The datasets [China Health and Retirement Longitudinal Survey (CHARLS)] for this study can be found at: https://opendata.pku.edu.cn/ (Peking University Open Research Data Platform).

## Author contributions

WL: conceptualization, supervision, and writing—review and editing. JK: conceptualization, methodology, data curation, investigation, software, writing—original draft, and visualization. FS: conceptualization, validation, formal analysis, writing—original draft, and writing—review and editing. All authors contributed to the article and approved the submitted version.

## Footnotes

^1^Specific policy documents are available on the official website of the Chinese Ministry of Human Resources and Social Security: http://www.mohrss.gov.cn/wap/zc/zcwj/201607/t20160705_242951.html.
