# Supplementary material for: Long-term care insurance and multidimensional poverty of middle-aged and elderly: Evidence from China
Source: Front Public Health. 2023 Feb 10;11:1100146. doi: 10.3389/fpubh.2023.1100146 (PMC9950558; doi:10.3389/fpubh.2023.1100146)
Supplement: Supplementary file 1 [file Presentation_1.pdf]

A Data Detail

A.1 *The sensitivity analysis of the impact of different k on IMD*

We decompose the IMD according to the classification criteria of urban and rural areas, age, and need for care, which is used to verify the robustness of our constructed IMD. Poverty is more prevalent among rural residents and those over 80 years of age than among urban residents and those under 80 years of age, as shown in Figure A1. Furthermore, the incidence of poverty is much higher for those who require care than for those who do not.

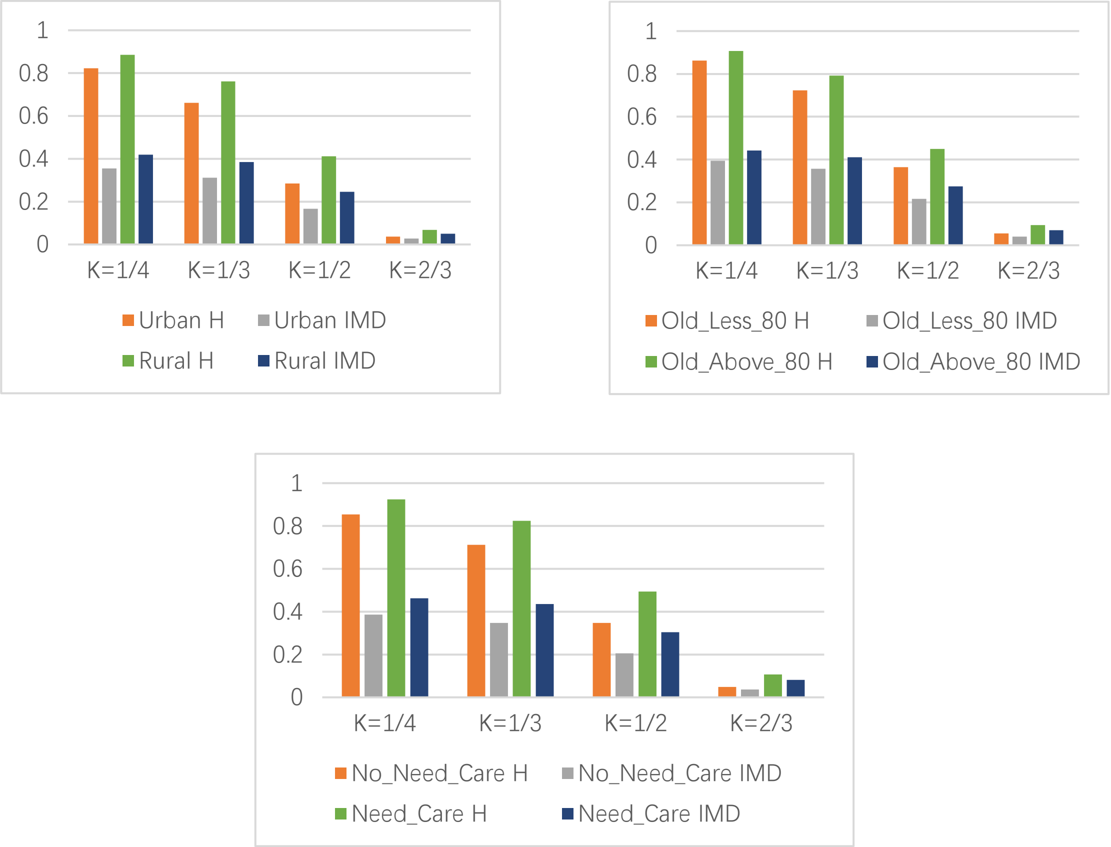

Figure A1. Decomposition of IMD by classification with different k

A.2 *The sensitivity analysis of the impact of different weighting structures on IMD*

We calculated the IMD for three different weight structures to see if different weight structures affect multidimensional poverty estimates. Regardless of the weight structure, the relative position of multidimensional poverty for each population group subgroup (e.g., urban/rural, age) should not change. The sensitivity analysis was carried out using three different weight structures. Weight 1 is an equal-weight assignment, weight 2 is an assignment in which individual rankings of each indicator are converted into weights and the average weight of individuals is calculated, and weight 3 is an assignment in which individual rankings of IMD are converted into weights. As shown

in Figure A2, the relative structure of the IMD remains unchanged when the weight structure changes, which proves the robustness of our measured IMD results. The rest of the analysis in this paper is based on a weighted structure with equal weights (weight 1).

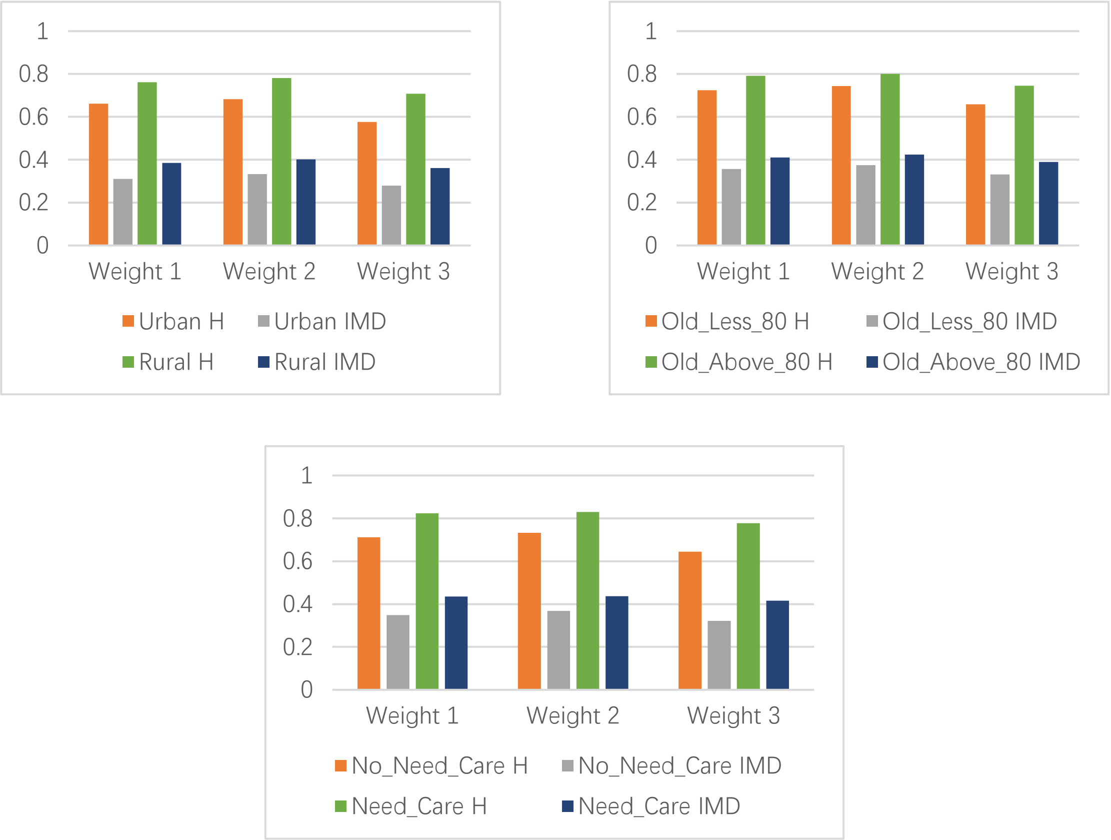

Figure A2. Decomposition of IMD by classification with different weight structures

B Timing of the LTCI Pilot

Table B1 shows when the LTCI pilots started in each city and lists the LTCI coverage groups for each pilot.

Table B1. Cities with LTCI pilots during the sample time

<table>
<tr><th colspan="3">Panel A</th></tr>
<tr><th colspan="3">LTCI pilot cities established by the central government</th></tr>
<tr><th>City</th><th>Date of LTCI Pilot</th><th>Coverage of People</th></tr>
<tr><td>Chengde*</td><td>2016.6</td><td rowspan="7">UEBMI</td></tr>
<tr><td>Qiqihar*</td><td>2016.6</td></tr>
<tr><td>Ningbo*</td><td>2016.6</td></tr>
<tr><td>Anqing*</td><td>2016.6</td></tr>
<tr><td>Guangzhou*</td><td>2016.6</td></tr>
<tr><td>Chongqing*</td><td>2016.6</td></tr>
<tr><td>Chengdu*</td><td>2016.6</td></tr>
<tr><td>Nantong</td><td>2016.6</td><td>UEBMI+URBMI</td></tr>
<tr><td>Suzhou*</td><td>2016.6</td><td rowspan="2">UEBMI+URRBMI</td></tr>
<tr><td>Shangrao*</td><td>2016.6</td></tr>
<tr><td>Qingdao*</td><td>The LTCI pilot was launched in 2012 among urban residents and began in January 2015 among rural residents.</td><td>2012 in UEBMI & 2015 in URRBMI & UEBMI</td></tr>
<tr><td>Jingmen*</td><td>2016.6</td><td>UEBMI+URRBMI</td></tr>
<tr><td>Shihezi</td><td>2016.6</td><td>UEBMI+URBMI</td></tr>
<tr><td>Shanghai*</td><td>2016.6</td><td>UEBMI+URRBMI(Residents aged 60 or older)</td></tr>
<tr><td>Changchun</td><td>Early self-pilot in May 2015</td><td>UEBMI+URBMI</td></tr>
</table>

Panel B		
Self-defined LTCI pilot cities in key provinces (i.e., Shandong, Jilin)		
City	Date of LTCI Pilot	Coverage of People
Weifang*	2015.1	UEBMI
Binzhou*	2018.1	UEBMI
Liaocheng*	2014.11	UEBMI
Dezhou	2018.12	UEBMI
Weihai	2018.7	UEBMI
Jilin*	2016.12	UEBMI+URBMI
Tonghua	2017.9	UEBMI+URBMI
Songyuan	2016.6	UEBMI

Panel C		
Self-implemented LTCI pilot cities		
City	Date of LTCI Pilot	Coverage of People
Julu, Xingtai	2016.8	Full coverage
Jiaxing	2017.6	UEBMI+URRBMI
Tonglu,Hangzhou	2017.3	UEBMI+URRBMI

Note: (1) UEBMI refers to Urban Employees' Basic Medical Insurance, URBMI refers to Urban Residents Basic Medical Insurance, and URRBMI refers to Urban and Rural Residents Basic Medical Insurance. (2) Cities with * are those in which the dataset was piloted with LTCI during the sample period. (3) Each wave of CHARLS data was interviewed from July-September. Therefore, the start date of the LTCI pilot in cities such as Binzhou is within the sample.

C Sensitivity Analyses

First, we convert the unbalanced panel dataset into a balanced panel dataset for DID estimation, and the results are detailed in column 1 of Table C1. It is discovered that random sample attrition does not affect the conclusions of this paper. Second, we replaced the outcome variable with the identification variable of multidimensional poverty and classified the respondents as being in multidimensional poverty or not. We use logit models to estimate the outcome variables. The results are shown in column 2 of Table C1. We find that LTCI reduces the likelihood of multidimensional poverty, income poverty, and subsistence consumption poverty significantly, while the DID estimates for material welfare poverty, social participation poverty, health poverty, and psychological welfare poverty remain negative but not statistically significant. Third, we convert time-varying DID to ordinary DID using data from the 2015 and 2018 waves. The results are detailed in column 3 of Table C1. The regression results are similar to the baseline regression, but the negative correlation between LTCI coverage and subsistence consumption poverty becomes statistically insignificant compared to the baseline regression. Fourth, we use the central government's second batch of LTCI pilot cities announced in 2020 as a control group; these cities implemented LTCI only four years later than the first batch, so it is reasonable to believe that these cities are more similar to the previous LTCI pilot cities. The CHARLS data includes seven of the second batch of LTCI pilot cities: Beijing, Tianjin, Hohhot, Fuzhou, Nanning, Kunming, and Hanzhong. These seven cities serve as the new control group. The estimated results are shown in column 4 of Table C1. We find that the estimation results are similar to the baseline regression. Finally, the results of the sensitivity analysis using IMD constructed with different K values as well as CSPI are shown in Table C2. We got similar results as with the baseline regression.

Table C1

Dependent variables	Coefficient on Treat × post			
	Balanced panel	logit	Ordinary DID	Replacement of control group
	(1)	(2)	(3)	(4)
IMD	-0.061***	-0.601*	-0.072**	-0.061**
	(0.020)	(0.320)	(0.033)	(0.022)
N	9888	36439	14836	5313
Income Poverty	-0.133***	-1.109**	-0.208**	-0.121**
	(0.041)	(0.486)	(0.082)	(0.047)
N	9888	36439	14836	5313
Living Consumption Poverty	-0.089	-1.086***	-0.128	-0.155*
	(0.083)	(0.358)	(0.094)	(0.078)
N	9888	36439	14836	5313
Material Well-being Poverty	-0.034	-0.171	-0.016	0.039
	(0.065)	(0.552)	(0.032)	(0.051)
N	9888	36439	14836	5313
Social Participation Poverty	-0.058	-0.241	-0.031	-0.062
	(0.072)	(0.326)	(0.053)	(0.063)
N	9888	36439	14836	5313
Health Poverty	-0.079	-0.457	-0.065	-0.081
	(0.057)	(0.359)	(0.065)	(0.062)
N	9888	36439	14836	5313
Psychological Well-being Poverty	0.029	-0.439	0.005	-0.012
	(0.026)	(0.553)	(0.042)	(0.041)
N	9888	36439	14836	5313

Note: ***, **, and * denote 1%, 5%, and 10% significance level, respectively. Except for the sixth column regression, all column regressions

control for year FE, individual FE, demographic and socioeconomic covariates, and household-level covariates, and the specific covariates controlled for are detailed in Table 3. Except for the second column regression, robust standard errors are clustered at the city level. The standard errors of the second column regressions are all derived from the bootstrap method.

Table C2

Dependent variables	Coefficient on Treat × post		
	K=1/2	K=2/3	CSPI
	(1)	(2)	(3)
IMD	-0.064***	-0.064***	
	(0.023)	(0.023)	
CSPI			-0.025**
			(0.011)
N	36439	36439	36439

Notes: Robust standard errors are clustered at the city level. ***, **, and * denote 1%, 5%, and 10% significance level, respectively. All regressions control for year FE, individual FE, demographic and socioeconomic covariates, and household-level covariates, as specified in Table 3.

D Robustness of Main Results

D.1 *Validity tests of the DID specification*

The validity of the DID strategy depends on the fact that there is no difference in the trend of change between the treatment and control groups before the onset of the policy. We test the parallel trend hypothesis using the event study method. As shown in Figure D1, the period treat on the x-axis indicates the year of reform, and the reference category is the year before LTCI (i.e., Pre1) and is omitted. The estimates for both the two and three pre-reform periods (i.e., Pre2 and Pre3) were not significant, suggesting that the trend of IMD change in the pre-reform treatment group was not different from that of the control group, implying that it passed the parallel trend hypothesis test. Furthermore, while IMD fell significantly in the year of the reform, LTCI did not significantly reduce poverty in the year following the reform.

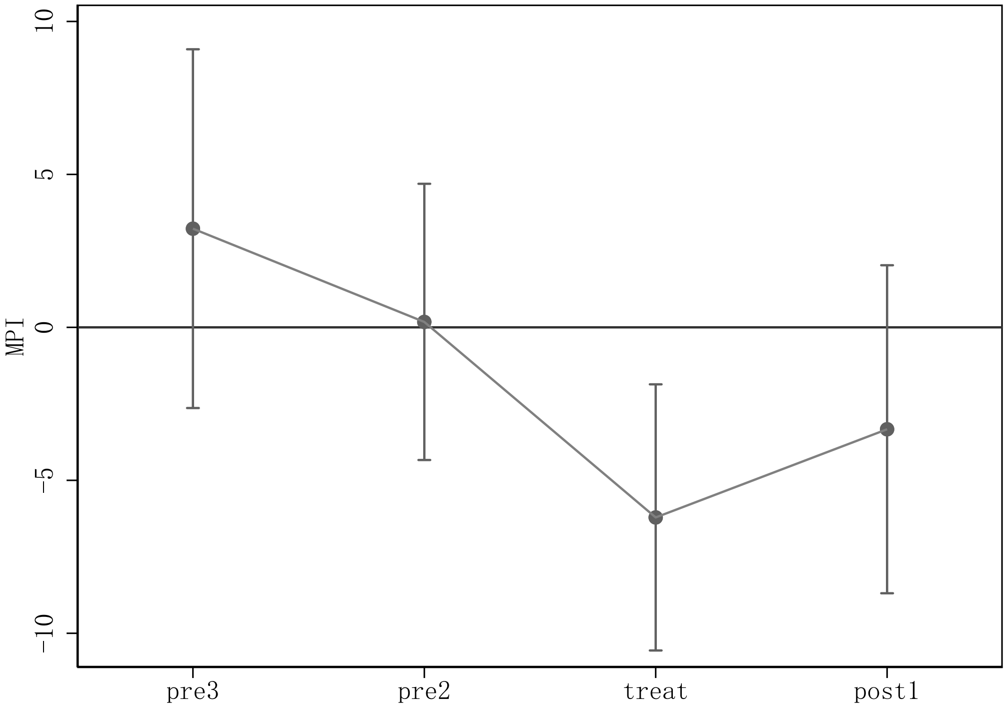

Figure D1. Event study for the effect of LTCI on IMD. *Notes:* The figure depicts the coefficients as well as the 95% confidence intervals for the event study analysis. On the *x*-axis, one period before the policy occurred (i.e., Pre1) is omitted as the reference group. The regressions include demographic and socioeconomic covariates, household-level covariates, year FE, and individual FE, with standard errors clustered at the city level.

We use Abadie SDID reweighted regression to further validate the plausibility of the parallel trend hypothesis in this paper. Abadie SDID reweighting regression requires two periods of balanced panel data and considering that the pilot cities in our data set started their pilots from 2012 to 2017, none of which had LTCI pilots in 2011 and all of which had LTCI pilots in 2018, we selected two periods of data from 2011 and 2018 to form a balanced panel for Abadie SDID reweighting regression. The regression results

are shown in Table D1, where we find that the estimated coefficients of the IMD and unidimensional poverty indicators are significantly negative, which further strengthens the credibility of the parallel trend hypothesis in this paper.

D.2 *Density distribution of sampled regression coefficients*

The density distribution of the 1000 sample regression coefficients for each outcome variable is shown in Figure D2. All sampled regressions are identical to the baseline regression, controlling for individual FE, year FE, demographic and socioeconomic covariates, and household-level covariates, with standard errors clustered at the city level. The curves represent the regression coefficient values after 1000 random sampling from the control group, and the vertical dashed line represents the baseline regression coefficient. We find that the baseline regression coefficients are very close to the median of the density function, confirming that the conclusions of this paper are not affected by the small proportion of sample size in the treatment group.

D.3 *Synthetic difference in difference (SDID)*

We use the Synthetic difference in difference (SDID) method to confirm that the small proportion of the sample in the treatment group does not affect the robustness of the findings in this paper. The SDID procedure calculates treatment effects by calculating the difference between treated and synthetic control individuals, with synthetic control individuals serving as the best weighting function for untreated individuals (individual weights) and pre-policy treatment period (time weights). The SDID estimator has many excellent properties, the most important of which is double robustness; given a correct, clearly set fixed effects model, the SDID estimator is robust under various weighting schemes; when the underlying fixed effects model is incorrectly set and the true data generation process involves a more general low-level data structure (e.g., a potential factor model), the SDID is also robust when the penalty weights for SC are appropriately skewed. As shown in the regression results for each outcome variable in Table D2, the small sample proportion of the treatment group in this paper does not affect the robustness of the study findings.

Table D1. Abadie SDID regression results

	IMD	Income Poverty	Living consumption Poverty	Material well-being Poverty	Social participation Poverty	Health Poverty	Psychological well-being Poverty
	(1)	(2)	(3)	(4)	(5)	(6)	(7)
Abadie SDID	-0.128***	-0.225***	-0.152***	-0.088***	-0.174***	-0.265***	-0.069**
	(0.016)	(0.028)	(0.034)	(0.030)	(0.050)	(0.052)	(0.028)
N	5151	5151	5151	5151	5151	5151	5151

Notes: Standard errors are in parentheses. The regressions include demographic and socioeconomic covariates and household-level covariates. The significance levels of 1%, 5%, and 10% are denoted by ***, **, and *, respectively.

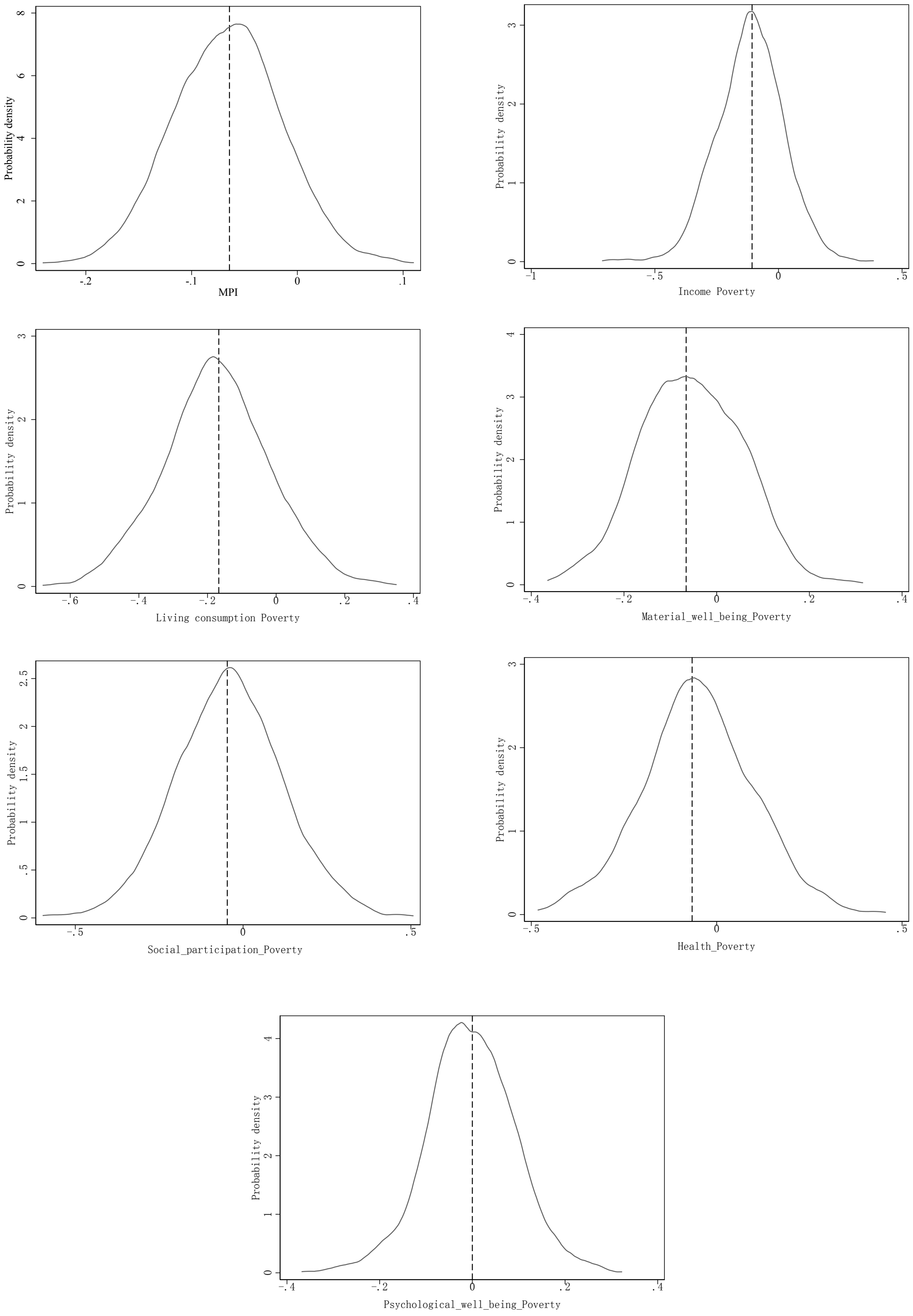

Figure D2. Density distribution of regression coefficients for each outcome variable

Table D2. Synthetic DID estimation results

Outcome Variables	Synthetic DID
IMD	-0.073**
	(0.034)
Income poverty	-0.162***
	(0.037)
Living consumption poverty	-0.095
	(0.091)
Material well-being poverty	-0.029
	(0.058)
Social participation poverty	-0.118
	(0.093)
Health poverty	-0.082
	(0.116)
Psychological well-being poverty	0.037
	(0.037)

Notes: Standard errors are in parentheses. Standard errors were calculated by bootstrap method. The significance levels of 1%, 5%, and 10% are denoted by ***, **, and *, respectively.

D.4 *A test of the random selection problem for policy pilots*

D.4.1 Controlling for the impact of urban characteristics over time

While controlling for individual FE and year FE in this paper, we control for the effects of city characteristics over time by incorporating city-year interactions to further mitigate the impact of city characteristics on city selection for the LTCI pilot. The regression results are detailed in Table D3. After incorporating the city-years interaction, we find that the conclusions of this paper remain robust.

Table D3. Regression results by incorporating city-years interactions

	Coefficient on Treat × post	
	DID	DID with matching
	(1)	(2)
Treat × post	-0.060***	-0.084***
	(0.021)	(0.031)
Age	-0.000	0.005
	(0.001)	(0.003)
Gender	0.024	-0.105*
	(0.022)	(0.061)
Marital status	0.013*	-0.003
	(0.008)	(0.025)
Hukou status	-0.013	-0.029
	(0.023)	(0.048)
Level of education(Reference group:No formal education (illiterate))		
Did not finish primary school but capable of reading or writing	-0.010	-0.019
	(0.007)	(0.017)
Sishu/home school	-0.059*	———
	(0.031)	———
Graduate from elementary school	-0.023***	-0.051***
	(0.009)	(0.019)
Graduate from middle school	-0.022*	-0.044*
	(0.011)	(0.025)
Graduate from high school	0.007	0.005

	(0.018)	(0.031)
Graduate from vocational school	-0.001	-0.032
	(0.022)	(0.040)
Graduate from Two/Three Year College / Associate degree	0.009	-0.220***
	(0.025)	(0.044)
Graduate from Four Year College / Bachelor's degree	-0.047	———
	(0.046)	———
Full-time employment	-0.006*	0.005
	(0.003)	(0.007)
Have basic old-age insurance	-0.012***	-0.005
	(0.003)	(0.009)
Retirement support(Reference group:Relying on children for retirement)		
Relying on savings for retirement	-0.004	0.000
	(0.006)	(0.026)
Relying on pension or retirement salary for retirement	0.002	0.015*
	(0.004)	(0.008)
Relying on commercial pension insurance for retirement	0.015	———
	(0.017)	———
Relying on others for retirement	0.023***	0.031*
	(0.005)	(0.017)
Have UEBMI	-0.012*	-0.013
	(0.007)	(0.018)
Have URBMI	0.002	0.057***
	(0.007)	(0.019)
Have URRBMI	0.003	0.023**

	(0.008)	(0.011)
Hospitalization	-0.001	-0.008
	(0.003)	(0.008)
Log (Inpatient out-of-pocket expenses + 1)	0.001	0.003
	(0.001)	(0.003)
Log (Household debt + 1)	0.000	0.002*
	(0.000)	(0.001)
Number of household members	0.005***	0.007**
	(0.001)	(0.003)
Number of household durables	-0.009***	-0.003*
	(0.001)	(0.002)
Household education & training expenses	-0.005***	-0.005***
	(0.000)	(0.001)
_cons	0.460***	0.234
	(0.053)	(0.186)
Observations	36439	11896
Adj-R^2	0.327	0.275
Individual FE	Y	Y
Year FE	Y	Y
City-Year FE	Y	Y

Notes: Robust standard errors are clustered at the city level. ***, **, and * denote 1%, 5%, and 10% significance level, respectively. All regressions control for year FE, individual FE, City-Year FE ,demographic and socioeconomic covariates, and household-level covariates, and the specific covariates controlled for are detailed in Table 2A.

D.4.2 Placebo test

We randomly selected 15 cities from the non-LTCI pilot cities as the pseudo-treatment group cities and continued to use the pilot time of the real treatment group cities for individual treatment group identification and DID strategy and the other cities as the control group cities. Since the pseudo-treatment group cities were randomly selected, the treatment effect of the pseudo-treatment group cities should theoretically not have a significant effect (i.e., the regression coefficient should theoretically be 0), and if the placebo test results found a significant treatment effect of the pseudo-treatment group cities, it indicates the presence of non-randomness factors or other policy factors.

We repeated the above regression procedure 500 times to avoid experimental errors interfering with the estimation results. All sampled regressions are identical to the baseline regression, controlling for individual FE, year FE, demographic and socioeconomic covariates, and household-level covariates, with standard errors clustered at the city level. Figure D3 depicts the density distribution of the estimated coefficients of each outcome variable after 500 repetitions of the regression, the curves indicate the regression coefficient values after 500 random sampling from non-pilot cities, and the vertical dashed lines indicate the baseline regression coefficients. It can be seen that the 500 regression coefficients of each outcome variable are concentrated and uniformly distributed around 0, whereas the estimated coefficients of each outcome variable's baseline regression deviate significantly from 0. As a result, the non-randomness in the selection process of LTCI pilot cities has no negative effects on the estimation results of this paper, and this further excludes the influence of other chance factors, enhancing the credibility of the paper's conclusions.

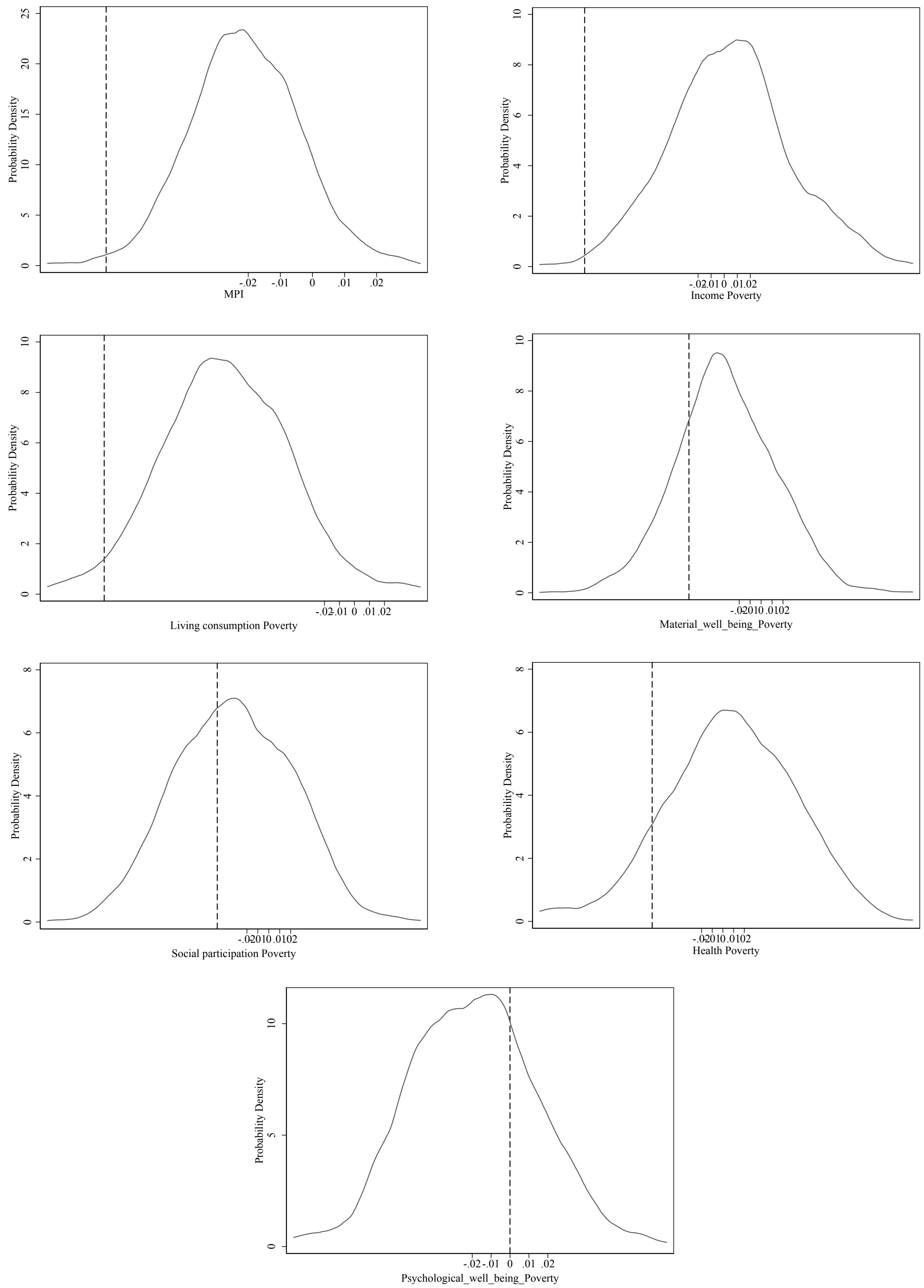

Figure D3. Placebo test: density distribution of regression coefficients for each outcome variable in the pseudo-treatment group

D.5 PSM-DID

First, we perform a balance test on the matching results. The results are shown in Table D4 & Table D5. Figure D4 illustrates the distribution of propensity scores before

and after matching.

Table D4. Year-by-year balance test before matching

	(1)<br>2013	(2)<br>2015	(3)<br>2018
Age	-0.0285	-0.0036	0.0091
	(-0.5955)	(-0.1352)	(0.6009)
Gender	-1.1973**	0.2744	-0.0598
	(-2.1516)	(0.6970)	(-0.2232)
Marital_status	0.0000	-0.7330	0.3374
	(.)	(-1.0725)	(1.4413)
Hukou_status	0.0000	0.9952	1.3406**
	(.)	(0.9384)	(2.2218)
1b.Level_of_education	0.0000	0.0000	0.0000
	(.)	(.)	(.)
2o.Level_of_education	0.0000		
	(.)		
3o.Level_of_education	0.0000	0.0000	0.0000
	(.)	(.)	(.)
4o.Level_of_education	0.0000		
	(.)		
5o.Level_of_education	0.0000		
	(.)		
6o.Level_of_education	0.0000		
	(.)		
7.Level_of_education	-0.4010		-0.2059
	(-0.6351)		(-0.3161)
8o.Level_of_education	0.0000	0.0000	
	(.)	(.)	
9o.Level_of_education	0.0000	0.0000	0.0000
	(.)	(.)	(.)
Full_time_employment	0.0000	-0.5022	-0.1860
	(.)	(-1.1750)	(-1.0505)
Have_basic_old_age_insurance	0.0000	0.0126	1.2930*
	(.)	(0.0142)	(1.6524)
1b.Retirement_support	0.0000	0.0000	0.0000
	(.)	(.)	(.)
2o.Retirement_support	0.0000	0.0000	
	(.)	(.)	
3o.Retirement_support	0.0000		
	(.)		
4o.Retirement_support	0.0000	0.0000	0.0000
	(.)	(.)	(.)

5o.Retirement_support	0.0000	0.0000	
	(.)	(.)	
Household_debt	0.0974*	-0.1232	0.0049
	(1.8099)	(-1.3926)	(0.1763)
Number_of_household_durables	0.1709*	0.1701	0.0272
	(1.9055)	(1.3805)	(0.1810)
Hospitalization	0.0000	0.7651***	0.0223
	(.)	(8.3031)	(0.1181)
Expenditure_on_education	0.0000	0.0370	0.0153
	(.)	(0.3040)	(0.3771)
Inpatient_out_of_pocket_expenses	0.0000	0.0000	-0.0430
	(.)	(.)	(-0.8243)
Number_of_household_members	-1.0950*	-0.1156	-0.1218
	(-1.7663)	(-0.7976)	(-0.5304)
2.Level_of_education		-1.7154***	0.2463
		(-3.1043)	(0.8305)
4.Level_of_education		-1.9773*	0.2133
		(-1.7434)	(0.4420)
5.Level_of_education		-1.2034	-0.4951
		(-0.8732)	(-1.1602)
6.Level_of_education		-1.6337	0.4726
		(-1.0106)	(0.8848)
7o.Level_of_education		0.0000	
		(.)	
3.Retirement_support		-0.2239	2.4284***
		(-0.1266)	(5.6645)
8.Level_of_education			0.1397
			(0.1915)
2.Retirement_support			0.9310
			(0.8126)
5.Retirement_support			1.2302*
			(1.6573)
N	45	4849	7002
r2_p	0.1606	0.0879	0.2098

Table D5. Year-by-year balance test after matching

	(1)	(2)
	2015	2018

Age	-0.0195	-0.0041
	(-0.6108)	(-0.2778)
Gender	0.3678	0.1245
	(0.5694)	(0.4114)
Marital_status	-0.8538	0.1883
	(-1.0738)	(0.5778)
Hukou_status	-0.7651	-0.0654
	(-1.3342)	(-0.1277)
2b.Level_of_education	0.0000	
	(.)	
4.Level_of_education	-0.6649	-0.1280
	(-1.0238)	(-0.2184)
5.Level_of_education	0.0740	-0.4157
	(0.0847)	(-0.7449)
6.Level_of_education	-0.7079	-0.1450
	(-0.5368)	(-0.2317)
7.Level_of_education	0.9790	-0.4765
	(1.3838)	(-0.6072)
Full_time_employment	-0.2290	0.0119
	(-0.4020)	(0.0506)
Have_basic_old_age_insurance	0.5175	-0.0387
	(0.5244)	(-0.0428)
1b.Retirement_support	0.0000	0.0000
	(.)	(.)
3.Retirement_support	-2.1796	0.1514
	(-1.2169)	(0.3341)
Household_debt	-0.0714	0.0218
	(-0.7710)	(0.7275)
Number_of_household_durables	0.1176	-0.0243
	(0.8380)	(-0.1799)
Hospitalization	0.8629**	0.0174
	(2.4607)	(0.0691)
Expenditure_on_education	0.0310	-0.0036
	(0.2435)	(-0.0780)
Inpatient_out_of_pocket_expenses	0.0000	-0.0334
	(.)	(-0.5551)
Number_of_household_members	-0.1652	-0.0173
	(-0.4652)	(-0.0662)
1b.Level_of_education		0.0000
		(.)
2.Level_of_education		-0.0698
		(-0.1939)
8.Level_of_education		-0.5056
		(-0.6106)

2.Retirement_support		-0.5020
		(-0.4304)
5.Retirement_support		-0.4882
		(-0.6267)
N	391	512
r2_p	0.1750	0.0081

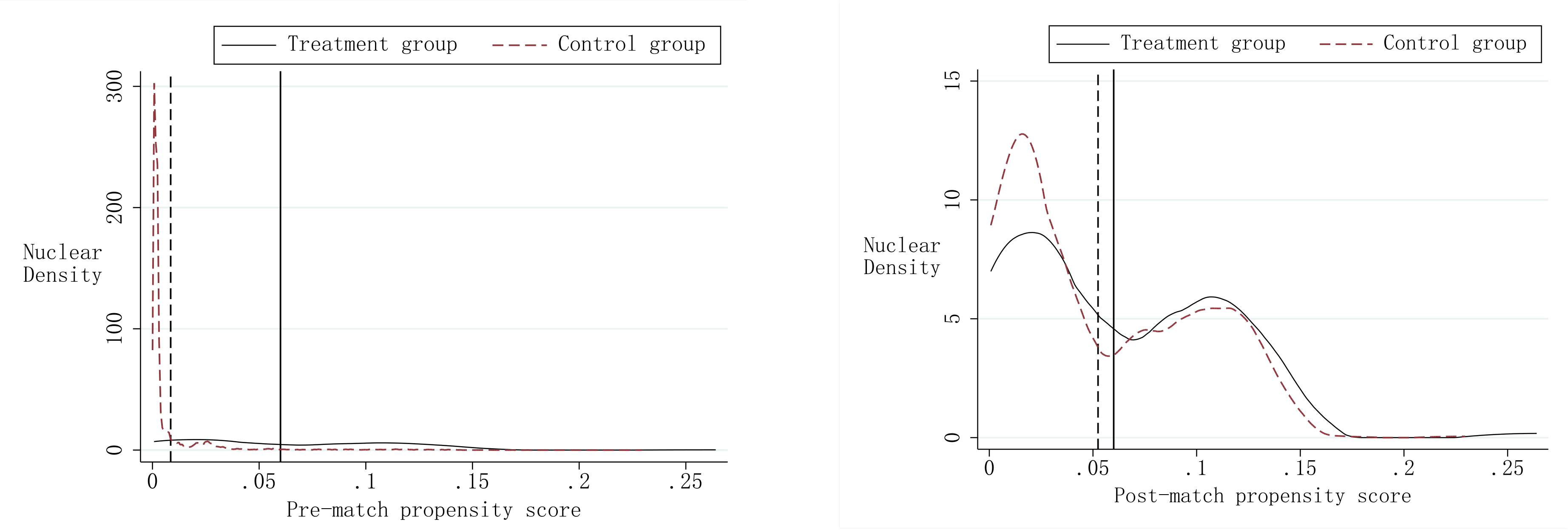

Figure D4. Distribution of propensity scores before and after matching

D.6 Bacon decomposition

The Bacon decomposition requires the dataset to be a balanced panel, and we further processed the dataset into a balanced panel to meet its requirements (the results of the sensitivity analysis indicate that this random sample attrition does not have a deleterious effect on the robustness of the conclusions, as detailed in column 1 of Table C1 in the Appendix). The results of the Bacon decomposition are shown in Table D6 & Figure D5. The results of the Bacon decomposition suggest that the possible presence of the "bad control group" in our DID estimation does not affect the robustness of the findings in this paper.

Table D6. Bacon Decomposition

	Beta	Total weight
Early_v_Late	-0.021	0.0010
Late_v_Early	0.046	0.0005
Never_v_timing	-0.071	0.9985

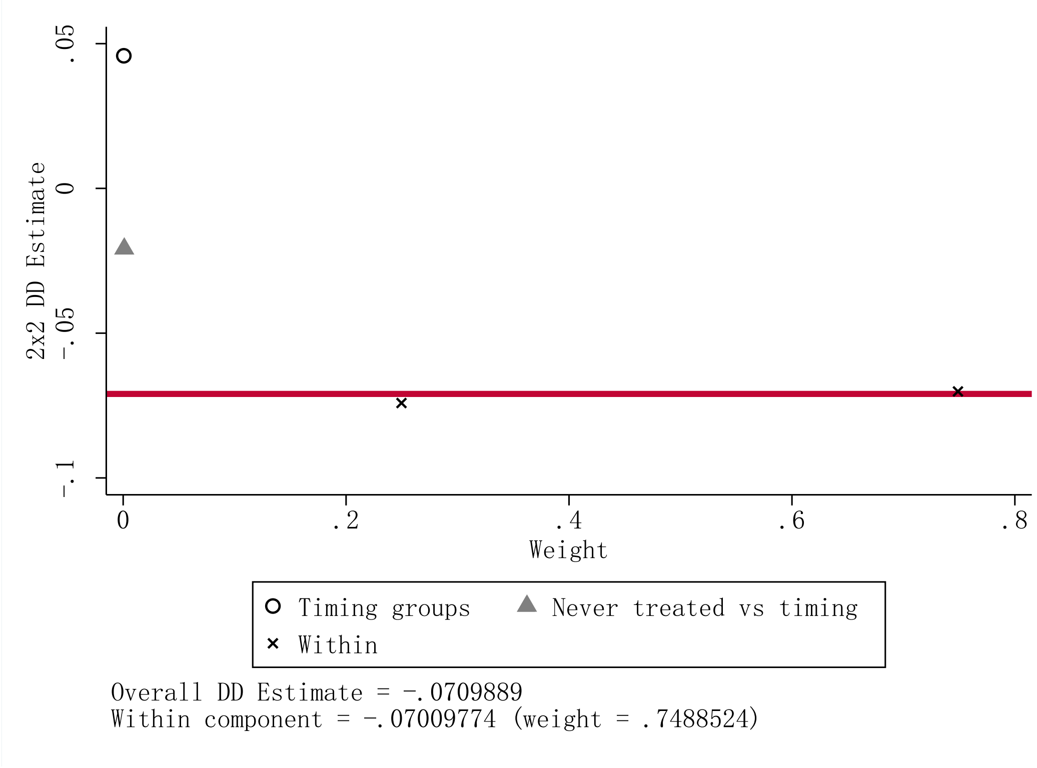

Figure D5. Bacon Decomposition

E Event study for the effect of LTCI on MPV

Figure E1. & Figure E2. depict the parallel trend hypothesis test results. The event study method revealed that the treatment and control groups had similar time trends prior to treatment, with LTCI having a significant negative effect on MPV in the current treatment period, more impact in the post-reform phase.

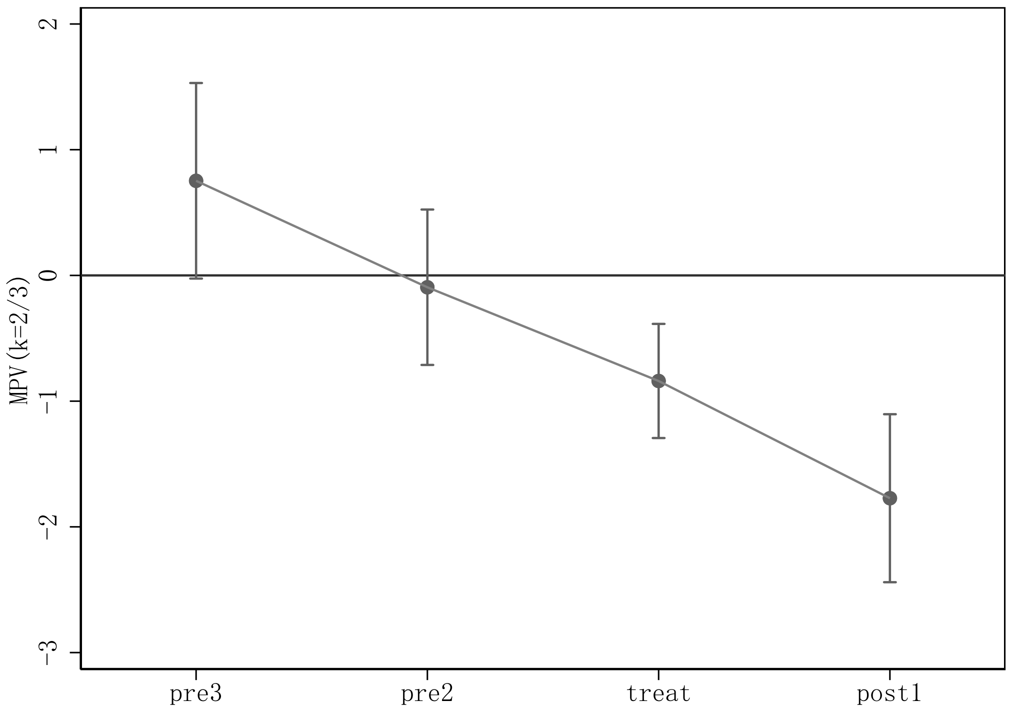

Figure E1. Event study for the effect of LTCI on MPV (k=2/3). *Notes:* The figure depicts the coefficients as well as the 95% confidence intervals for the event study analysis. On the *x*-axis, one period before the policy occurred (i.e., Pre1) is omitted as the reference group. The regressions include demographic and socioeconomic covariates, household-level covariates, year FE, and individual FE, with standard errors clustered at the city level.

Figure E2. Event study for the effect of LTCI on MPV (k=1/3). *Notes:* The figure depicts the coefficients as well as the 95% confidence intervals for the event study analysis. On the *x*-axis, one period before the policy occurred (i.e., Pre1) is omitted as the reference group. The regressions include demographic and socioeconomic covariates, household-level covariates, year FE, and individual FE, with standard errors clustered at the city level.

F Heterogeneity

Table F1. Effect of LTCI on individuals in different quartiles of IMD

	(1)<br>25%<br>quantile	(2)<br>50%<br>quantile	(3)<br>75%<br>quantile	(4)<br>90%<br>quantile	(5)<br>25%<br>quantile	(6)<br>50%<br>quantile	(7)<br>75%<br>quantile	(8)<br>90%<br>quantile
LTCI_Duration					-0.053*	-0.064***	-0.075***	-0.080***
					(0.028)	(0.019)	(0.025)	(0.031)
LTCI_Coverage	-0.053*	-0.064***	-0.075***	-0.080***				
	(0.028)	(0.019)	(0.025)	(0.031)				
Age	-0.000	-0.000	0.000	0.000	-0.000	-0.000	0.000	0.000
	(0.002)	(0.001)	(0.001)	(0.002)	(0.002)	(0.001)	(0.001)	(0.002)
Gender	0.020	0.022	0.024	0.025	0.020	0.022	0.024	0.025
	(0.031)	(0.021)	(0.028)	(0.035)	(0.031)	(0.021)	(0.028)	(0.035)
Marital_status	0.020*	0.014*	0.008	0.005	0.020*	0.014*	0.008	0.005
	(0.012)	(0.008)	(0.010)	(0.013)	(0.012)	(0.008)	(0.010)	(0.013)
Hukou_status	-0.023	-0.015	-0.007	-0.004	-0.023	-0.015	-0.007	-0.004
	(0.033)	(0.022)	(0.030)	(0.037)	(0.033)	(0.022)	(0.030)	(0.037)
1b.Level_of_education	0.000	0.000	0.000	0.000	0.000	0.000	0.000	0.000
	(0.000)	(0.000)	(0.000)	(0.000)	(0.000)	(0.000)	(0.000)	(0.000)
2.Level_of_education	-0.017	-0.012	-0.007	-0.005	-0.017	-0.012	-0.007	-0.005
	(0.012)	(0.008)	(0.010)	(0.013)	(0.012)	(0.008)	(0.010)	(0.013)
3.Level_of_education	-0.076	-0.071*	-0.066	-0.063	-0.076	-0.071*	-0.066	-0.063
	(0.061)	(0.040)	(0.054)	(0.068)	(0.061)	(0.040)	(0.054)	(0.068)
4.Level_of_education	-0.028*	-0.024***	-0.021*	-0.019	-0.028*	-0.024***	-0.021*	-0.019
	(0.014)	(0.009)	(0.013)	(0.016)	(0.014)	(0.009)	(0.013)	(0.016)
5.Level_of_education	-0.026	-0.022*	-0.017	-0.015	-0.026	-0.022*	-0.017	-0.015
	(0.017)	(0.012)	(0.016)	(0.019)	(0.017)	(0.012)	(0.016)	(0.019)
6.Level_of_education	0.004	0.008	0.012	0.013	0.004	0.008	0.012	0.013
	(0.027)	(0.018)	(0.024)	(0.030)	(0.027)	(0.018)	(0.024)	(0.030)
7.Level_of_education	0.017	-0.002	-0.022	-0.031	0.017	-0.002	-0.022	-0.031
	(0.036)	(0.024)	(0.032)	(0.040)	(0.036)	(0.024)	(0.032)	(0.040)

8.Level_of_education	-0.006	0.005	0.017	0.022	-0.006	0.005	0.017	0.022
	(0.041)	(0.027)	(0.037)	(0.046)	(0.041)	(0.027)	(0.037)	(0.046)
9.Level_of_education	0.007	-0.040	-0.086	-0.107	0.007	-0.040	-0.086	-0.107
	(0.114)	(0.076)	(0.102)	(0.127)	(0.114)	(0.076)	(0.102)	(0.127)
Full_time_employment	-0.006	-0.005	-0.005	-0.004	-0.006	-0.005	-0.005	-0.004
	(0.005)	(0.003)	(0.004)	(0.005)	(0.005)	(0.003)	(0.004)	(0.005)
Have_basic_old_age_insurance	-0.017***	-0.015***	-0.013***	-0.012**	-0.017***	-0.015***	-0.013***	-0.012**
	(0.004)	(0.003)	(0.004)	(0.005)	(0.004)	(0.003)	(0.004)	(0.005)
1b.Retirement_support	0.000	0.000	0.000	0.000	0.000	0.000	0.000	0.000
	(0.000)	(0.000)	(0.000)	(0.000)	(0.000)	(0.000)	(0.000)	(0.000)
2.Retirement_support	-0.006	-0.003	-0.000	0.001	-0.006	-0.003	-0.000	0.001
	(0.009)	(0.006)	(0.008)	(0.010)	(0.009)	(0.006)	(0.008)	(0.010)
3.Retirement_support	0.001	0.003	0.005	0.007	0.001	0.003	0.005	0.007
	(0.006)	(0.004)	(0.005)	(0.006)	(0.006)	(0.004)	(0.005)	(0.006)
4.Retirement_support	0.023	0.020	0.016	0.015	0.023	0.020	0.016	0.015
	(0.025)	(0.017)	(0.022)	(0.028)	(0.025)	(0.017)	(0.022)	(0.028)
5.Retirement_support	0.021**	0.023***	0.026***	0.027***	0.021**	0.023***	0.026***	0.027***
	(0.009)	(0.006)	(0.008)	(0.010)	(0.009)	(0.006)	(0.008)	(0.010)
Household_debt	0.000	0.000	0.000	0.000	0.000	0.000	0.000	0.000
	(0.001)	(0.000)	(0.000)	(0.001)	(0.001)	(0.000)	(0.000)	(0.001)
Number_of_household_durables	-0.008***	-0.008***	-0.009***	-0.009***	-0.008***	-0.008***	-0.009***	-0.009***
	(0.001)	(0.001)	(0.001)	(0.001)	(0.001)	(0.001)	(0.001)	(0.001)
Hospitalization	-0.001	-0.001	-0.001	-0.001	-0.001	-0.001	-0.001	-0.001
	(0.006)	(0.004)	(0.005)	(0.006)	(0.006)	(0.004)	(0.005)	(0.006)
Expenditure_on_education	-0.005***	-0.005***	-0.006***	-0.006***	-0.005***	-0.005***	-0.006***	-0.006***
	(0.001)	(0.000)	(0.001)	(0.001)	(0.001)	(0.000)	(0.001)	(0.001)
Inpatient_out_of_pocket_expenses	0.001	0.001	0.001	0.001	0.001	0.001	0.001	0.001
	(0.001)	(0.001)	(0.001)	(0.001)	(0.001)	(0.001)	(0.001)	(0.001)
Number_of_household_members	0.004***	0.005***	0.006***	0.006***	0.004***	0.005***	0.006***	0.006***
	(0.002)	(0.001)	(0.001)	(0.002)	(0.002)	(0.001)	(0.001)	(0.002)
Have_UEBMI	-0.014	-0.012*	-0.011	-0.010	-0.014	-0.012*	-0.011	-0.010
	(0.010)	(0.007)	(0.009)	(0.011)	(0.010)	(0.007)	(0.009)	(0.011)

Have_URBMI	-0.004	0.001	0.007	0.009	-0.004	0.001	0.007	0.009
	(0.012)	(0.008)	(0.011)	(0.013)	(0.012)	(0.008)	(0.011)	(0.013)
Have_URRBMI	-0.004	0.002	0.008	0.011	-0.004	0.002	0.008	0.011
	(0.009)	(0.006)	(0.008)	(0.010)	(0.009)	(0.006)	(0.008)	(0.010)
0b.year	0.000	0.000	0.000	0.000	0.000	0.000	0.000	0.000
	(0.000)	(0.000)	(0.000)	(0.000)	(0.000)	(0.000)	(0.000)	(0.000)
1.year	0.057***	0.052***	0.048***	0.046***	0.057***	0.052***	0.048***	0.046***
	(0.006)	(0.004)	(0.005)	(0.006)	(0.006)	(0.004)	(0.005)	(0.006)
2.year	0.076***	0.072***	0.069***	0.067***	0.076***	0.072***	0.069***	0.067***
	(0.008)	(0.005)	(0.007)	(0.009)	(0.008)	(0.005)	(0.007)	(0.009)
3.year	0.063***	0.065***	0.068***	0.069***	0.063***	0.065***	0.068***	0.069***
	(0.012)	(0.008)	(0.011)	(0.013)	(0.012)	(0.008)	(0.011)	(0.013)
N	36439	36439	36439	36439	36439	36439	36439	36439

Table F2. Heterogeneous effects of LTCI

	IMD	Income Poverty	Living consumption Poverty	Social participation Poverty	Material well-being Poverty	Health Poverty	Psychological well-being Poverty
	(1)	(2)	(3)	(4)	(5)	(6)	(7)
Panel A. By rural/urban residents							
LTCI Duration × rural	-0.020	-0.032	-0.105***	0.015	0.022	-0.027	-0.034***
	(0.016)	(0.043)	(0.021)	(0.017)	(0.018)	(0.035)	(0.013)
LTCI Duration × urban	-0.037***	-0.064***	-0.096*	-0.034	-0.040**	-0.031	0.022
	(0.013)	(0.016)	(0.050)	(0.030)	(0.016)	(0.029)	(0.023)
Panel B. By fiscal subsidies							
LTCI Duration × no fiscal subsidies	-0.022**	-0.054**	-0.061***	0.003	-0.006	-0.039	-0.010
	(0.010)	(0.025)	(0.021)	(0.024)	(0.016)	(0.029)	(0.020)
LTCI Duration × fiscal subsidies	-0.064***	-0.058***	-0.215***	-0.091***	-0.070**	-0.002	0.054
	(0.017)	(0.008)	(0.047)	(0.008)	(0.032)	(0.044)	(0.054)

Panel C. By eligibility assessment							
LTCI Duration × only serve	-0.031***	-0.051**	-0.098***	-0.022	-0.021	-0.027	0.006
	(0.012)	(0.020)	(0.036)	(0.026)	(0.017)	(0.027)	(0.023)
LTCI Duration × moderate+serve	-0.063***	-0.205***	-0.103***	0.065***	-0.046***	-0.137***	-0.013
	(0.005)	(0.013)	(0.015)	(0.012)	(0.016)	(0.014)	(0.008)
Panel D. By service mode							
LTCI Duration × only institutional care	-0.037***	-0.200***	-0.242***	0.328***	-0.050***	-0.035***	-0.029***
	(0.005)	(0.012)	(0.012)	(0.014)	(0.013)	(0.013)	(0.008)
LTCI Duration × home care+ institutional care	-0.032***	-0.053***	-0.097***	-0.024	-0.022	-0.030	0.006
	(0.012)	(0.019)	(0.036)	(0.026)	(0.017)	(0.026)	(0.023)

Panel E. By reimbursement							
LTCI Duration × low pay	0.000	-0.012	-0.018	-0.017	-0.028	0.029	0.057
	(0.013)	(0.032)	(0.020)	(0.051)	(0.038)	(0.042)	(0.035)
LTCI Duration × high pay	-0.047***	-0.075***	-0.136***	-0.021	-0.019	-0.058***	-0.018
	(0.011)	(0.012)	(0.043)	(0.029)	(0.016)	(0.016)	(0.018)
N	36439	36439	36439	36439	36439	36439	36439

Notes: Robust standard errors are clustered at the city level. ***, **, and * denote 1%, 5%, and 10% significance level, respectively. All regressions control for year FE, individual FE, demographic and socioeconomic covariates, and household-level covariates, as specified in Table 3.
